# Cognitive functioning associated with acute and subacute effects of classic psychedelics and MDMA - a systematic review and meta-analysis

**DOI:** 10.1038/s41598-024-65391-9

**Published:** 2024-06-26

**Authors:** Lukas A. Basedow, Tomislav Majić, Nicklas Jakob Hafiz, Engi A. E. Algharably, Reinhold Kreutz, Thomas G. Riemer

**Affiliations:** 1 Department of Psychology, Clinical Psychology and Psychotherapy, Philipps-Universität Marburg, Gutenbergstraße 18, 35037 Marburg, Germany; 2 Psychedelic Substances Research Group, Department of Psychiatry and Neurosciences, Charité-Universitätsmedizin Berlin, Berlin, Germany; 3 Department of Psychiatry und Neurosciences, Charité-Universitätsmedizin Berlin, Corporate Member of Freie Universität Berlin and Humboldt-Universität zu Berlin, Berlin, Germany; 4 Institute for Educational Quality Improvement (IQB), Humboldt-Universität zu Berlin, Berlin, Germany; 5 Charité – Universitätsmedizin Berlin, corporate member of Freie Universität Berlin, Humboldt-Universität zu Berlin, and Berlin Institute of Health, Institute of Clinical Pharmacology and Toxicology, Charité Campus Mitte, Charitéplatz 1, 10117 Berlin, Germany

**Keywords:** Cognition, Psychedelic, MDMA, Acute, Subacute, Afterglow, Human behaviour, Cognitive neuroscience, Medical research

## Abstract

Classic psychedelics and MDMA have a colorful history of recreational use, and both have recently been re-evaluated as tools for the treatment of psychiatric disorders. Several studies have been carried out to assess potential long-term effects of a regular use on cognition, delivering distinct results for psychedelics and MDMA. However, to date knowledge is scarce on cognitive performance during acute effects of those substances. In this systematic review and meta-analysis, we investigate how cognitive functioning is affected by psychedelics and MDMA during the acute drug effects and the sub-acute (“afterglow”) window. Our quantitative analyses suggest that acute cognitive performance is differentially affected by psychedelics when compared to MDMA: psychedelics impair attention and executive function, whereas MDMA primarily affects memory, leaving executive functions and attention unaffected. Our qualitative analyses reveal that executive functioning and creativity may be increased during a window of at least 24 h after the acute effects of psychedelics have subsided, whereas no such results have been observed for MDMA. Our findings may contribute to inform recommendations on harm reduction for recreational settings and to help fostering differential approaches for the use of psychedelics and MDMA within a therapeutic framework.

## Introduction

Classic psychedelics (“psychedelics”), such as lysergic acid diethylamide (LSD) or psilocybin, can facilitate a variety of psychological effects, including perceptual distortions, cognitive restructuring, mood changes, changes in meaning of percepts, and dissolution of bodily boundaries^1^. These effects are believed to be mainly induced via agonism at the serotonin (5HT) 2a receptor^2^. In addition to the specifics of acute effects, psychedelics are unique regarding their ability to exert psychological effects that by far outlast their acute physiological presence^3–6^. Temporal dynamics of psychedelics can therefore be differentiated into acute (psychedelic states), sub-acute, and long-term effects^5–7^.

Notably, even regular high-frequent use of psychedelics has not been associated with persisting, long-term impairment of cognitive functioning^8,9^. In contrast, research on how psychedelics affect cognition during the acute and subacute period of drug effects has not yet revealed a clear pattern^10^. Nevertheless, knowledge on the acute effects of psychedelics on cognition appears to be essential for the estimation of the risk profile in recreational use, and subsequent recommendations for harm reduction^11,12^. Moreover, knowledge on acute and subacute cognitive effects is also important regarding potential therapeutic applications of psychedelics in the treatment of psychological conditions like depression or anxiety within a psychotherapeutic framework. For instance, there is preliminary evidence that the quality and intensity of *acute* subjective experiences during psychedelic states might predict long-term treatment outcomes^13–15^. Moreover, with regard to the *subacute* (“afterglow”) period, an increased effectivity for therapeutic interventions has anecdotally been reported^16–18^.

Even if the entactogen 3,4-methylenedioxymethamphetamine (MDMA) does not belong to the group of classic psychedelics, it has sometimes also been referred to as a “psychedelic” in the context of substance-assisted therapy^19,20^. Despite serotonergic neurotransmission being a shared pathway of action, psychedelics and MDMA differ in their pharmacodynamic^2,21,22^, toxicological^23–25^, and phenomenological^19,26^ properties. Additionally, psychedelics and MDMA also diverge in their effects beyond the stage of acute intoxication, including sub-acute and long-term effects. Psychedelics, in particular, have been shown to induce sustained sub-acute effects^7^ not currently detected in MDMA use. Furthermore, psychedelic use appears to be mostly non-toxic in terms of neuropsychological functioning^8,9^, while research in MDMA users often suggest persistent cognitive deficits, particularly in memory functioning^27–31^. However, these MDMA-related findings remain disputed, with some authors arguing that reported differences are more likely to stem from pre-morbid differences rather than substance-induced cognitive dysfunction^32,33^, and may be less pronounced than initially reported^33,34^. Nevertheless, the literature suggests that the repeated use of psychedelics and MDMA is related to distinct neuropsychological outcomes.

To our knowledge to date there is no data investigating the relationship between the acute cognitive effects of those substances and the longitudinal differences between psychedelics and MDMA. Meanwhile, comprehensive understanding of the acute cognitive effects of psychedelics and MDMA appears to be crucial for various areas of research and for the evaluation of acute harms associated with those substances. Considering the increasing recreational use of psychedelics and MDMA^35–37^, providing accurate harm reduction resources and information about potential acute effects becomes highly relevant. The cognitive effects of psychoactive substances, especially those consumed recreationally without medical supervision, play an important role in the determination of their safety profiles. In comparison, GABAergic substances like alcohol and benzodiazepines lead to reduced reaction times^38–40^ and vigilance^41,42^ while also increasing risk-taking behaviour^43^, and potentially inducing delusions of sobriety^44^. On the other hand, stimulant drugs, e.g. caffeine or amphetamine, may increase attentional performance^45,46^ or induce hypervigilance^47^, while similarly inducing a tendency to overestimate capabilities under the influence^48,49^. Thus, the acute cognitive effects of psychoactive substances may have consequences for issues such as road safety, performance enhancement or reduction, or social interactions. To properly evaluate the effects of psychedelics and MDMA in these domains, a comprehensive assessment of their acute cognitive effects is needed.

In addition to prevention and harm reduction, the acute and subacute cognitive effects of psychedelics and MDMA may also play a role in their use as therapeutic agents. Given that higher levels of cognitive functioning are related to better outcomes and treatment success in traditional psychotherapy^50–54^, it is reasonable to assume it plays an important role in the context of psychedelic-assisted therapy (PAT) as well. Moreover, while not part of the commonly-applied PAT framework, psychedelics have been used therapeutically in the form of microdoses (1/10th of a common dose, e.g., 10µg of LSD)^55,56^. In this context, psychedelics stemming from the black market are often used by patients for self-medication of attention deficit hyperactivity disorder^57^ (ADHD). This type of use, along with harm reduction aspects, considerations regarding PAP and other instances of psychedelic and MDMA use outside of trial contexts (e.g., in recreational or retreat settings), highlights the need for an up-to-date analysis of acute cognitive effects.

To provide this analysis we conducted a systematic review and meta-analysis of the acute cognitive effects of psychedelics and MDMA. Our study goes beyond previous research by aiming to include analyses of the acute cognitive effects of microdosing, the cognitive post-acute effects of psychedelics, and indirect comparisons of the acute effects of psychedelics and MDMA.

## Method

### Search strategy

We performed electronic searches on PubMed, Web of Science, and Embase, from the respective database inception to July 10th, 2023, following PRISMA guidelines. In addition, we searched the clinical trial registries ClinicalTrials.gov and EudraCT. The search in PubMed, Web of Science, and Embase utilized an algorithm that combined terms related to psychedelics or MDMA with terms associated with cognitive testing in an iterative manner (see Supplementary Table S1). In the trial registries, individual names of psychedelics and MDMA were used for the search. References were retrieved through electronic searches and by manual searches of the reference lists of selected articles. Articles published in English, German, French, Serbian or Spanish were included. Detailed in- and exclusion criteria are described in the Supplementary Method Section.

### Data extraction

Two authors (LAB, TGR) independently screened all search results, with input from a third author (TM) in cases of disagreement regarding inclusion or exclusion. Additionally, the two raters classified all studies into acute or sub-acute assessments.

### Study quality

We assessed study quality and risk of bias using the Cochrane risk of bias (RoB) rating scales^58^ for parallel-arm trials and cross-over trials. For sequentially designed trials, we used the rating tool from the National Heart, Lung, and Blood Institute (NHLBI)^59,60^. LAB and TGR independently rated study quality, and a consensus was reached through discussion for each parameter.

### Meta-analysis

We included studies that reported results as mean with standard deviations into the meta-analyses and performed analyses if at least three viable studies per domain were included. As many studies reported multiple tests per domain we performed a three-level meta-analysis^61^ on the standardized mean difference, incorporating between-study as well as within-study heterogeneity into the model. We modeled the effect sizes as nested within their respective studies by assigning a nested random effect to the grouping variable “study”. Thus, the random intercept was allowed to vary across different studies. Test statistics and confidence intervals for the fixed effects used a t-distribution. The test of moderators checks the assumption that ${\mu }_{mdma}={\mu }_{psychedelics}=0$, which is a test for an overall effect of the variable “drugType” (MDMA vs. psychedelics). To compare both drug types we defined a contrast, testing if ${\mu }_{mdma}-{\mu }_{psychedelics}=0$ (Wald-Type Test).

Separate analyses were conducted for studies with sub-acute assessments (testing done up to 24h after drug administration) and microdosing approaches. Heterogeneity was measured using the I^2^ statistic. For studies reporting multiple dosages and/or times of administration, data for the highest dosage and closest time to peak drug concentration (t_max_; based on comparison values from^62–64^) were selected. We planned six sensitivity analyses: (1) restricting included studies to accuracy-based test or (2) speed-based test), (3) excluding the study with the highest weight, (4) using measurements closest to drug administration instead of closest to t_max_ in cases of multiple measurements, (5) restricting to the same psychedelic, (6) excluding studies with a high risk of bias rating. Statistical analyses were carried out using R version 4.3.2^65^ and the following R packages: dmetar v. 0.1.0^66^, metafor v. 4.4.0^67^. All used packages are cited in the Supplementary Materials.

## Results

### Study selection

**Figure 1:**
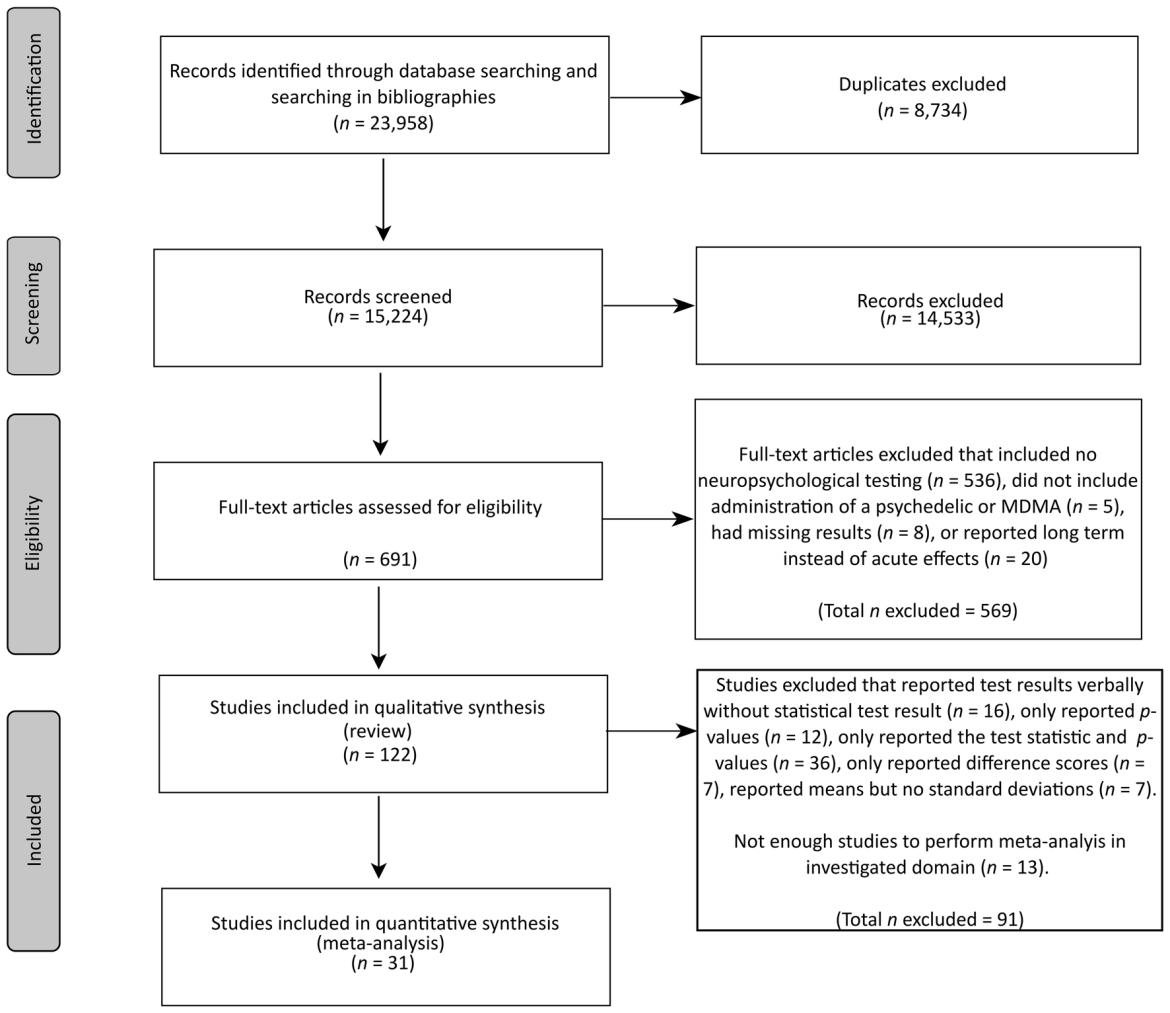
PRISMA flowchart.

Excluding duplicates, our search yielded 15,224 records, out of which 691 were selected for full-text screening. Following full-text screening, 122 records were included in the systematic review, and 31 records were viable for quantitative analysis. There was high inter-rater agreement (Fleiss’ kappa k = 0.98; 95%CI [0.97–0.99]), with only ten cases of 691 screened full-texts involving disagreements. Two of these disagreements were related to the type of investigated substances, in which cases the third reviewer (TM) provided expertise. The other eight disagreements were due to the unclear nature of the included tests and were concluded based on literature research and expertise by reviewer TGM. Supplementary Tables S2–9 provide an overview of the included studies. Specifically, 68 studies investigated the cognitive effects of classic full-dose psychedelics (Supplementary Tables S2 and S6), 34 studies focused on MDMA (Supplementary Tables S3 and S7), nine studies explored microdosing (Supplementary Tables S4 and S8), and nine studies assessed cognition within 24 h after acute dosing (Supplementary Tables S5 and S9). Figure 1 (and Supplementary Table S11) displays details of the different phases of the search.

### Quality rating

All 83 studies that were assessed using the Cochrane RoB tool, including both parallel-arm as well as cross-over studies, were rated as having a high risk of bias. Out of the 37 studies assessed with the NHLBI instrument, 34 were considered to be of poor quality, and three were rated as fair quality (see Supplementary Tables S2–5). The high risk of bias in the included studies is mainly due to the unblinding effects of psychedelics and MDMA^68^. If proper blinding would have been accomplished, five sequential studies would have been rated fair, while 41 placebo-controlled studies would have been rated as having some concern and 5 studies as having a low risk of bias. For the purpose of the respective sensitivity analyses, we excluded the blinding criterion from the quality rating, allowing to control for differences in other sources of bias risk between studies.

### Qualitative analysis

Here, we present the results regarding the influences of psychedelics and MDMA on memory performance, executive functioning, and attention. Additional findings on other cognitive abilities, such as visuospatial skills, intelligence, motor skills, and language functions, are provided in the supplement.

#### Full-dose, acute assessment of psychedelics

Out of the 67 studies investigating the acute effects of full-dose psychedelics, 55 studies (*n* = 1062) specifically assessed memory, executive function, or attention. Supplementary Table S2 provides an overview of study design, participants, and tests administered. Cognitive tests were conducted between 10^69^ and 360^70,71^ min after drug administration, with four studies^70–73^ involving repeated assessments. LSD dosages ranged from 40 µg^74^ to 2 µg/kg^75^, psilocybin dosages ranged from 45^76^ to ~ 430 µg/kg^71^, DMT dosages ranged from 0.17^69^ to 0.32 mg/kg^77,78^, one study administered 100 ml of ayahuasca^79^, and ten studies involved multiple dosages^70,71,73,74,76–78,80–82^.

##### Memory

A total of 21 studies (*n* = 553) investigated memory performance under the influence of ayahuasca^79^, DOM^83,84^, LSA^72^, LSD^74,75,82,85–92^, 2C-B^93^ and psilocybin^71–73,93–96^, utilizing 22 distinct memory tests. The majority of studies (1 LSA, 3 psilocybin, 6 LSD, 1 DOM)^72–74,82,84,85,88–90,95,96^ did not show any acute effects of psychedelics on memory across fifteen tasks. On the other hand, eleven studies (4 psilocybin, 1 2C-B, 6 LSD, 1 ayahuasca)^71,72,75,79,82,86,87,91–94^ demonstrated impaired memory performance across twelve tasks, while only one study (DOM)^83^ using one task showed improved memory performance.

##### Executive functioning

Twenty-four studies (*n* = 503) applied 27 distinct tasks to assess the acute effects of Ayahuasca^79,97,98^, 2C-B^93^, LSD^80,81,87–89,99–107^, mescaline^108^ and psilocybin^71,93,109–112^ on executive functioning. Sixteen of the included studies (1 2C-B, 6 psilocybin, 10 LSD)^71,81,87,88,93,99–104,107,109–112^ reported impaired executive functions across 19 tasks, while twelve studies (2 psilocybin, 3 ayahuasca, 7 LSD)^71,79,80,87,89,97–99,101,105,106,111^ reported no effects across 15 tasks. Additionally, five studies (2 ayahuasca, 2 LSD, 1 mescaline)^79,97,99,101,108^ showed improved performance across six tests.

##### Attention

Twenty-three studies (*n* = 291) investigated the acute effects of DMT^69,77,78,113^, 2C-B^93^, mescaline^114^, LSD^74,80,81,90,115–118^, and psilocybin^70,76,93,95,109,119–123^, utilizing 18 distinct attention tasks. Among these studies, fifteen (2 DMT, 1 2C-B, 10 psilocybin, 3 LSD)^69,70,76,77,93,95,109,116–123^ reported impaired attentional performance in 17 tasks, while nine studies (2 DMT, 6 LSD, 1 mescaline)^74,78,80,81,90,113–116^ reported no effect across eleven tasks.

#### Full-dose, acute assessment of MDMA

Among the 34 studies on MDMA, 33 studies (*n* = 716) examined its effects of MDMA on memory, executive functioning, and attention, as summarized in Supplementary Table S3. Cognitive tests were administered between 15 min^124^ and 11 h^125,126^ min after drug administration, with three studies^124–126^ involving repeated assessments. Dosages used ranged from 25 mg^127^ to 1.7 mg/kg^128,129^, with five studies involving multiple dosages^125–127,130,131^.

##### Memory

Twenty-two studies (*n* = 454)^124–127,131–147^ on MDMA examined its effect on memory using 25 different tests. Out of these studies, sixteen^124,126,131–140,142,143,146–148^ concluded that MDMA acutely impairs memory across 23 tasks, while twelve studies^124,127,132–134,137,140–142,144,145,149^ showed no effect in 13 tasks. In addition, one study^133^ reported improved performance in one measure.

##### Executive functioning

Thirteen studies (*n* = 240)^125,127,129,130,135,137,140,141,144,150–153^ investigated the effects of acute MDMA exposure on executive functioning, utilizing 14 different tasks. The majority of these studies^125,127,129,130,135,137,144,150–153^ showed no significant effect of MDMA on fourteen tasks, while four studies^130,140,141,152^ reported impaired performance across four tasks.

##### Attention

Thirteen studies (*n* = 270)^21,124,125,127,128,133,135,141,144,147,153–155^ assessed the effects of MDMA on attention using 18 different tests. The majority of these studies^21,124,125,127,128,133,141,144,147,153–155^ found no effect of MDMA on attentional performance across 17 measures, while four studies^125,135,141,153^ reported impaired attention in four tasks.

#### Microdose, acute assessment of psychedelics

Eight studies (*n* = 209) conducted investigations on the cognitive effects of psychedelic microdosing^156–163^ on memory, executive functioning, and attention, as detailed in Supplementary Table S4. The studies utilized LSD^157–159,161–163^, psilocybin-containing truffles^156^, and psilocybin-containing mushrooms^160^.

##### Memory

Four of these studies (*n* = 124)^157,158,161,162^ examined the effects of microdosed LSD on memory using five tests, and none of them found any significant effect.

##### Executive functioning

Five studies (*n* = 161) evaluated the effects of microdoses of LSD^157,161,163^ and psilocybin^156,160^ on executive functioning using nine different tasks. Among them, two studies^160,163^ reported impaired performance in two tasks, one study^156^ reported improved performance in two tasks, while the majority of studies^157,160,161,163^ found no significant difference across eleven tasks.

##### Attention

Four of the reviewed studies (*n* = 106) assessed attentional performance under the influence of a LSD^158,159,163^ or psilocybin^160^ microdoses using six different tasks. Three of these studies^158,160,163^ found no significant effect on attentional performance across five tasks, while one study^159^ reports reduced attention in one task.

#### Full-dose, sub-acute assessment of psychedelics

Eight studies (*n* = 282)^4,99,164–169^ had their participants perform cognitive tasks of memory, executive functioning, or attention on the day after consumption of a psychedelic substance (Supplementary Table S5).

##### Memory

Three studies (*n* = 46)^166,167,170^ reported sub-acute effects of LSD on five different memory tasks. Two of these studies^166,170^ showed increased memory performance across three tasks, and two studies ^167,170^ showed no effect in two task.

##### Executive functioning

Six studies (*n* = 288) evaluated sub-acute executive functioning using five different tasks in psilocybin^165^, ayahuasca^4,164,168^, 5-Meo-DMT^167,169^, and LSD^170^. Out of these studies, two^164,170^ showed impaired performance in two tasks, while four studies^4,165,168,169^ reported improvement across four measures, and three studies^4,167,170^ found no effect in four tasks.

##### Attention

Two studies (*n* = 46)^167,170^ investigated sub-acute effects of LSD using two tasks and observed no significant difference in performance compared to placebo.

#### Full-dose, sub-acute assessment of MDMA

Two studies (*n* = 27)^144,171^ assessed the subacute cognitive effects of MDMA and found no effect on three memory tasks, three executive functioning tasks and one attention task (see Supplementary Table S5).

### Quantitative analysis

#### Memory

**Figure 2:**
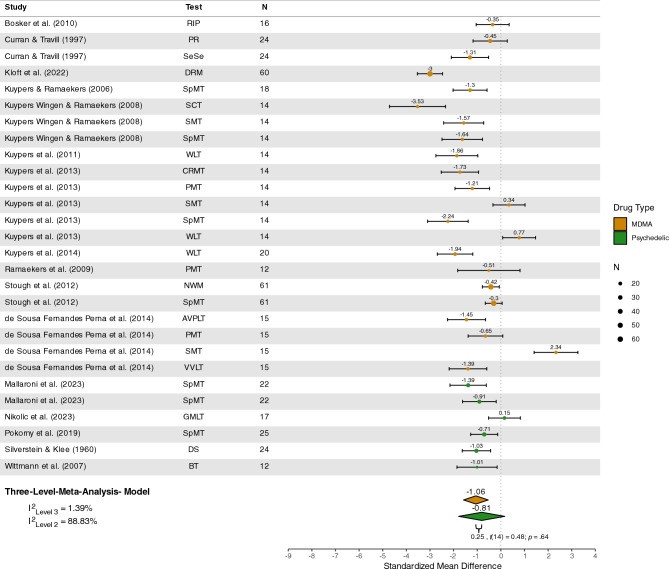
Forest plot of psychedelic and MDMA studies involving memory tasks. *MDMA,* 3,4-methylenedioxymethamphetamine; *RIP*, rapid information processing; *PR*, prose recall; *SeSe*, serial sevens; *VVLT*, visual verbal learning task; *PMT*, prospective memory task; *SMT*, Sternberg memory task; *AVPLT*, abstract visual pattern learning task; *DRM*, Deese/Roediger-McDermott word list; *SpMT*, spatial memory task; *SCT*, star counting task; *WLT*, word learning task; *CRMT*, continuous recognition memory task; *NWM*, numeric working span; *GMLT*, Groton maze learning task; *SpWM*, spatial working memory task; *DS*, digit span; *BT*, block tapping.

Sixteen studies^73,75,87,93,96,126,127,132,133,136,138–141,143,148^ were included in the quantitative analysis of memory tasks, with a total of *n* = 368 participants. Memory was not significantly impaired under psychedelics (*Z* = − 0.81 [95%CI − 1.78; 0.17], *p* = 0.10) but under MDMA (*Z* = − 1.06 [95% CI − 1.58; − 0.54], *p* < 0.001), see Figure 2. Furthermore, no difference in memory performance was detected between these substances (*Z* = 0.25 [95% CI − 0.86; 1.35] *p* = 0.64). The estimated variance components were τ2_Level3_ = 0.02 and τ2_Level2_ = 1.09. Therefore I^2^_Level3_ = 1.39% of total variation could be attributed to between-study, and I^2^_Level2_ = 88.83% to within-study heterogeneity.

#### Executive functioning

**Figure 3:**
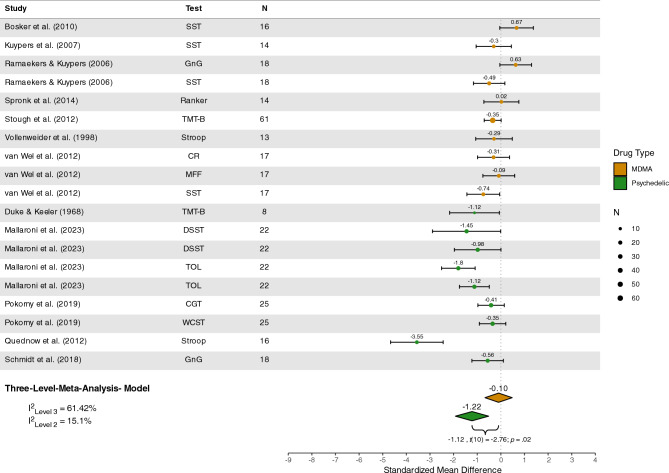
Forest plot of psychedelic and MDMA studies involving executive functioning tasks. *MDMA,* 3,4-methylenedioxymethamphetamine; *SST*, stop-signal task; *DT*, discounting task; *GnG*, go/no-go task; *TMT-B*, trail-making task trial B; *MFF*, matching familiar figures; *CR*, cue-reversal task; *TOL*, Tower of London; *DSST*, digit-symbol substitution; *WCST*, Wisconsin card sorting task; *CGT*, Cambridge gambling task.

Twelve studies^87,93,100,109,110,125,127,129,130,141,151,152^ with a total of *n* = 242 participants were included in the quantitative analysis of executive functioning. Meta-analyses revealed significant impairment under psychedelics (*Z* = − 1.22 [95%CI − 1.92; − 0.52], *p* = 0.003), but not under MDMA (*Z* = − 0.10 [95% CI − 0.67; 0.48], *p* = 0.72), with a significant group difference observed (*Z* = − 1.12 [95% CI − 2.03; − 0.22], *p* = 0.02), see Figure 3. The estimated variance components were τ2_Level3_ = 0.31 and τ2_Level2_ = 0.08 Therefore

I^2^_Level3_ = 61.42% of total variation could be attributed to between-study, and I^2^_Level2_ = 15.10% to within-study heterogeneity.

#### Attention

**Figure 4:**
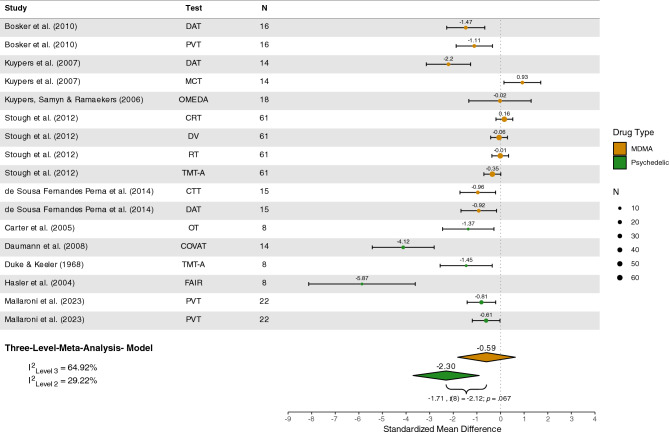
Forest plot of psychedelic and MDMA studies involving attention tasks. *MDMA,* 3,4-methylenedioxymethamphetamine; *DAT*, divided attention task; *PVT*, psychomotor vigilance task; *CTT*, critical tracking task; *OMEDA*, object movement under divided attention; *MCT*, Macworth clock task; *RT*, simple reaction time; *DV*, digit vigilance; *CRT*, choice reaction time; *TMT-A*, trail-making task trial A; *OT*, object tracking; *COVAT*, covert orienting of attention test; *FAIR*, Frankfurt attention inventory.

Ten studies^69,76,93,95,109,125,127,133,141,155^ encompassing *n* = 184 participants were included in the quantitative analysis of attention. Significant impairment was detected under psychedelics (*Z* = − 2.30 [95%CI − 3.71; − 0.90], *p* = 0.005) but not MDMA (*Z* = − 0.59 [95%CI − 1.82; 0.63], *p* = 0.30), see Figure 4. However, subgroup analyses indicated no significant difference in attentional performance under psychedelics compared to MDMA (*Z* = − 1.71 [95%CI − 3.57; 0.15], *p* = 0.07). The estimated variance components were τ2_Level3_ = 1.05 and τ2_Level2_ = 0.47. Therefore I^2^_Level3_ = 64.92% of total variation could be attributed to between-study, and I^2^_Level2_ = 29.22% to within-study heterogeneity.

#### Microdosing

**Figure 5:**
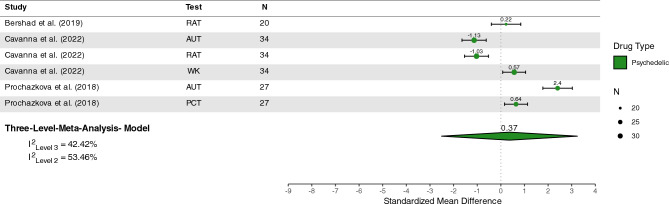
Plot of microdosing studies involving creativity tasks. *LSD,* lysergic acid diethylamide; *RAT,* remote association task; *AUT,* alternative uses task; *WK*, Wallach-Kogan Test; *PCT,* picture concept task.

Only three microdosing could be included for quantitative analysis and these studies were exclusively related to the domain of creativity. Thus, no quantitative analysis of microdosing effects on other cognitive domains could be performed. Three studies^156,157,160^, involving *n* = 81 participants, were included in a quantitative analysis of creativity tasks under microdose conditions. The overall effect estimate revealed no significant effect of psychedelic microdoses on creativity tasks (*Z* = 0.37 [95%CI − 2.51; 3.24], *p* = 0.64), see Figure 5. The estimated variance components were τ2_Level3_ = 0.76 and τ2_Level2_ = 0.96 Therefore 

I^2^_Level3_ = 42.42% of total variation could be attributed to between-study, and I^2^_Level2_ = 54.46% to within-study heterogeneity.

### Sensitivity analyses

An overview of all completed sensitivity analyses can be found in Supplementary Table S10.

When limiting studies to speed-based tasks the significant effects of MDMA memory disappeared (*Z* = 1.43 [95%CI − 12.87; 10.01], p = 0.36). Similarly, the effect of psychedelics on executive functioning is non-significant in speed-based tasks (*Z* = − 1.54 [95%CI − 3.63; 0.55], *p* = 0.10) and when limiting the study selection to LSD (*Z* = − 0.43 [95%CI − 2.63; 1.78], *p* = 0.25) or psilocybin (*Z* = − 2.11 [95%CI − 5.12; 0.91], *p* = 0.09) alone. Regarding attention, the significant effect of psychedelics only persists when excluding the study with highest weight (*Z* = − 2.92 [95%CI − 4.65; − 1.18], *p* = 0.006), but not for any other sensitivity analysis, see Supplementary Table S10.

## Discussion

In our analysis of the acute and subacute effects of classic psychedelics and MDMA on cognition, we report the following findings: acute effects of classic psychedelics are consistently associated with reduced cognitive performance across multiple domains. Our qualitative and quantitative results indicate that the most pronounced effects are observed in the domain of attention, whereas memory and executive functioning remain less intensively affected. In contrast, MDMA primarily impacts acute memory performance. Notably, the included studies were highly heterogeneous in terms of the tests applied, reported outcomes of each test, drug dosages, and time between drug and test administration, which hinders accurate comparisons across studies. Our results provide important information in the context of psychedelic harm reduction, for instance, when it comes to choosing a safe setting for recreational use, considering to drive a car or to elsewise participate in road traffic. In addition, impaired memory functions under MDMA might potentially reduce certainty in users about the amount of consumed MDMA which could lead to dangerous redosing and overdosing^172^.

Our results also provide some insight when it comes to fostering therapeutic interventions for the framework of psychedelic-assisted therapy (PAT). Specifically, during the acute experience, applying cognitive interventions might be challenging, if patients are unable to properly follow the therapist’s guidance. In contrast, with psychedelics specifically, there might be a place for increased therapeutic support during the days after psychedelic dosing sessions, since cognitive performance is potentially increased during that subacute (‘afterglow’) window. For MDMA, on the other hand, the effects on memory performance might actually be beneficial in the context of Posttraumatic Stress Disorder (PTSD)-specific therapy that involves reconsolidation of traumatic memories under the influence of MDMA^173^. The reduced ability to encode, especially emotional^149^, information might reduce reconsolidation of traumatic memories and promote extinction, thus promoting a reduction of PTSD symptoms^174^. In fact, there is already a line of research focused on producing psychopharmacological agents that can modify memory to support PTSD therapy^175^. Thus, our results support the notion that psychedelic and MDMA-assisted therapy are two distinct forms of therapies that are applicable to different disorders and different treatment approaches, as reflected in their distinct cognitive effects facilitating distinct therapeutic interventions. In addition to the consistent evidence that altered reconsolidation of memories is a main mechanism of MDMA-assisted therapy^173,176^, the re-experiencing of memories might be relevant in therapy with classic psychedelics as well^177,178^.

The disparity between psychedelics and MDMA with regard to acute cognitive effects may be linked to differences in acute subjective effects. In short, while psychedelic experiences might be characterized by strong perceptual distortions^26,76,179^ and potentially distracting experiences such as reduced self-other boundaries^180^, MDMA experiences are often marked by an pronounced emotional pattern of effects^26,181^. The emotional effects of MDMA may impair the ability to retrieve state-incongruent memories (e.g., retrieving neutral stimuli such as word lists, in a highly aroused positive emotional state)^149,182,183^ and as such lead to the observed memory deficits, while the effects of psychedelics might provide sensory distraction that reduces acute attention. Notably, under microdosing conditions which lack the perceptual alterations of psychedelics^184^, those effects on cognition disappear^156,159,160,163^. While this may suggest that the nature of the psychedelic alterations might be inherently distracting, this could also be the result of dose-dependent effects, as studies with multiple dosages of psilocybin showed dose-dependent effects on cognitive performance^71,73,73,76^.

While it is unclear if the observed difference is due to these factors, there is strong evidence indicating a reduction in attention under psychedelics which aligns with the recently proposed model of cognitive functioning under psychedelics by Sayalı and Barrett^185^. This model posits that psychedelics induce a transient increase of cognitive flexibility while simultaneously impairing attentional capacities. As we found preliminary evidence that attention was significantly reduced when focusing on speed-based tasks but not accuracy-based tasks, our findings support the notion that psychedelics specifically reduce sustained attention or vigilance but not cognitive control (as is needed for accuracy-based tasks). Additional confirmatory evidence is provided by research regarding creative performance under the influence of psychedelics^99,111,186^ and animal research^187,188^ showing an increased ability to switch action patterns under psilocybin.

Furthermore, this model of increased cognitive flexibility aligns with results regarding neuropsychological consequences of long-term psychedelic use. For instance, there is no evidence for lasting cognitive deficits^8,9^, but some preliminary evidence indicating potential sustained cognitive benefits beyond the acute psychedelic effects. Our qualitative review indicated that executive functioning and creativity may be increased within a window of 24 h after a using a psychedelic^4,165,168,169^. Furthermore, studies have shown improvements in executive functioning one week after psilocybin administration^189^, and there is as some evidence of improved executive performance in individuals who regularly use ayahuasca^190,191^. Finally alterations in neuronal networks of executive control^192^, salience and default mode networks^193^ have been reported, as well as increased neuroplasticity for up to one week after administration^194^. As for MDMA, only few studies have investigated the sub-acute effects, and the evidence so far indicates no impairments or improvements on cognition^144,171^. Even though, animal research indicates that MDMA might enhance critical periods of social learning in the days after administration^195,196^, there is also evidence linking MDMA-induced memory deficits to alterations in serotonergic functioning^197–199^, which is in line with findings indicating deteriorations in the serotonergic system after repeated MDMA use^200^. Neurobiological long-term consequences of repeated use might also account for the proposed long-term cognitive deficits associated with MDMA use^27^. However, while neuronal adaptive processes that occur as a result of MDMA use are consistently reported, the significance and relevance of these changes for cognitive alterations remain a topic of ongoing debate^34^.

## Limitations

First, the included studies reported results from various cognitive tests and employed heterogeneous research methods, including different dosages or times of drug administration. This heterogeneity limits the robustness of the presented results and reduces the certainty of our conclusion. However, we addressed this limitation by selecting the highest dosage and dosage closest to suspected peak effects for the meta-analysis and by combining multiple cognitive tests to load on the same factor, facilitating quantitative analysis (as is commonly done in other fields^201^). This enables us to draw conclusions despite methodological variety, although these conclusions should be considered as preliminary. Future studies should take care to report common protocols to allow for analysis of different phases of cognitive processing under psychedelics (e.g.^183^).

Secondly, the chosen methods did not allow for more fine-grained analyses of dose-dependent or time-dependent effects of psychedelics or MDMA on cognition. Future studies should aim to assess the effects of a broader range of psychedelic dosages (from micro- to macro-dosing) and across multiple timepoints.

Thirdly, we did not analyze the contextual setting of the psychedelic/MDMA session or the test administration. Since psychedelic effects are known to be highly influenced by context^202^, variations in the settings of the experience itself may have impacted the effects on cognition.

Fourth, blinding participants and researchers in studies involving the acute administration of psychoactive substances is a well-known challenge^68^. As indicated in the risk of bias assessment, most of the included studies did not achieve full blinding, which may have introduced strong expectancy effects, reducing the validity of the reported results. However, unlike in clinical studies, it remains unclear what participants' expectations are in cognitive assessments under the influence of psychoactive substances and how these expectations might influence actual test performance.

Fifth, we were not able to incorporate analyses taking into account previous drug exposure of participants. While most studies reported if their participants had ever used the investigated substance, only a fraction reported use frequency in a fashion that would allow for co-variate analysis. This lack of data could be an additional factor contributing to the high heterogeneity found in our results, especially considering the chronic tolerance reported by MDMA users^27^.

Sixth, while we reviewed a large number of studies in a qualitative manner only few studies could be included for quantitative analysis. Thus, our quantitative results are not representative of the literature at large but are based on a few select studies. Future studies in this field should take care to report as much of the raw data as possible to support more overarching analysis efforts like ours.

Finally, all included studies were conducted with healthy volunteers and did not include patient populations. As the effects of psychotropic medications sometimes can vary between the healthy and patient populations^203^, the same may be true for psychedelics and MDMA. Further studies are needed to investigate the cognitive effects of psychedelics and MDMA within the framework of psychedelic-assisted therapy.

## Conclusion

This study is the first to contain a meta-analytic assessment of the acute cognitive effects of classic psychedelics, including a comparison with the effects of MDMA. Additionally, this is the largest synthesis of neuropsychological data for psychedelics as well as MDMA so far conducted. We report robust evidence that psychedelics acutely reduce attention, and preliminary evidence that psychedelics may also have acute detrimental effects on memory and executive functioning. In contrast, MDMA appears to have the strongest impact on memory performance, whereas attention and executive functions are less intensively affected in the acute phases. Conversely, during the sub-acute (‘afterglow’) period when acute effects have worn off, psychedelics potentially exert beneficial effects on executive functioning, while such effects have not been found for MDMA. Our findings add neuropsychological evidence for fostering distinct therapeutic approaches for psychedelics when compared to MDMA-assisted therapy. Given deteriorations of attention under psychedelics, psychotherapeutic interventions might be less fruitful during acute effects of the substance when compared to the subacute “afterglow” period. In contrast, given the specifics of acute effects of MDMA on cognition, psychotherapeutic techniques may be more fruitful during the acute experience when compared to psychedelics, whereas therapy during the subacute window might be somewhat less effective. In sum, there is a need for more research regarding cognitive underpinnings of classic psychedelics and MDMA with respect to prevention and for their use as tools for psychotherapy.

### Supplementary Information

Supplementary Information.

## Data Availability

The datasets used and analyzed during the current study are available in the GitHub repository: https://github.com/nickhaf/MDMA_Psychedelics_meta.

## Code availability

The code for the analysis and the corresponding data are available in the GitHub repository: https://github.com/nickhaf/MDMA_Psychedelics_meta.
