# Supplementary material for: Cognitive functioning associated with acute and subacute effects of classic psychedelics and MDMA - a systematic review and meta-analysis
Source: Sci Rep. 2024 Jun 26;14:14782. doi: 10.1038/s41598-024-65391-9 (PMC11208433; doi:10.1038/s41598-024-65391-9)
Supplement: Supplementary file 1 — Supplementary Information. [file 41598_2024_65391_MOESM1_ESM.docx]

SUPPLEMENT - Cognitive functioning associated with acute and subacute effects of classical psychedelics and MDMA - a systematic review and meta-analysis

**Supplemental Method Section**

**Criteria for selection**

The following criteria were used to select articles.

**Population.** Studies with healthy participants and patient studies were included.

**Intervention.** To be included a paper had to be either a qualitative study dealing with cognitive abilities of intoxicated psychedelic or MDMA users, an experimental study in which subjects received a psychedelic or MDMA and underwent cognitive assessment, or an observational study that performed cognitive assessment on people self-administering a psychedelic or MDMA.

**Comparisons.** Studies were selected if they compared the tests scores of people under the influence of a psychedelic or MDMA, with either a placebo group, a baseline performance measure, a sober control group, or normative data.

**Outcomes.** To be selected the article had to report the results of at least one measure, assessing at least one of the following cognitive domains: memory, executive function, attention, visuospatial abilities, intelligence, verbal functioning, motor functions.

**Study type.** For the assessment of acute effects, qualitative studies of acute psychedelic or MDMA intoxication, or experimental human studies in which psychedelics or MDMA were administered, published in peer-reviewed journals were included. We excluded animal research, review papers, opinion pieces, letters to the editors, and books.

**Search algorithm**

While we are confident that the search algorithm (see Supplemental Table S1) led to a complete oversight of the literature, there is a possibility that we missed publications that did not use any of the included terms but still reported neuropsychological test results.

**Meta-Analysis**

We used R version 4.3.2^1^ and the following R packages: dmetar v. 0.1.0^2^, extrafont v. 0.19^3^, ggbrace v. 0.1.0^4^, ggtext v. 0.1.2^5^, grid v. 4.3.2^1^, here v. 1.0.1^6^, metafor v. 4.4.0^7^, psych v. 2.3.12^8^, renv v. 1.0.3^9^, rmarkdown v. 2.25^10–12^, rstatix v. 0.7.2^13^, tidyverse v. 2.0.0^14^.

**Supplemental Results Section**

**Qualitative analysis of visuo-spatial abilities, general intelligence, verbal functioning and motor functions**

*Psychedelics*

Eleven studies^15–25^ investigated the effects of psychedelic on seven different visuo-spatial tasks across *n* = 305 participants. Nine studies^15,16,18–21,23–25^ found an impairment in seven tasks, while three studies^15,17,22^ reports no effect in three task. Eight studies ^19–21,26–30^ investigated the effects of psychedelic general intelligence (*n* = 163), all of which found impairment in five measures, while^27^ also reports no effect on two measures. Six studies (*n* = 147)^22,27,31–34^ included ten measures of verbal functioning and showed impairment in five of those, while the other five measures, were not affected. Six studies^17,29,35–37^ (*n* = 51) investigated motor functions in seven tasks and found impaired performance in six of those.

*MDMA*

Three studies^38–40^ across *n* = 62 participants investigated visuo-spatial abilities under the influence of MDMA across three tasks and only^38^ report impairment in one task. Four studies^41–44^ used seven tasks to investigate verbal functioning in *n* = 80 participants, with^42^ and^44^ showing impairment in two tasks. Five studies^40,45–48^ (*n* = 74) assessed motor functions in five tasks, and^46^ showed impaired performance in one of those, while^47^ showed improved performance in two tasks. No studies assessed general intelligence under MDMA influence.

*Microdosing*

One study ^49^ showed no effect of microdosing on general intelligence while one study ^50^ showed increased verbal functioning in one of two tasks. No studies assessed motor functions or visuo-spatial abilities under microdosing conditions.

*Sub-acute effects*

Two studies investigated sub-acute visuo-spatial abilities ^51,52^ in two tasks, with ^52^ reporting improved performance in one of the two. One study ^51^ showed increased sub-acute verbal functioning in one task. No studies investigated sub-acute performance in intelligence or motor tasks.

Supplemental Table S1: Included search terms related to STs and cognitive testing

| (MDMA OR ecstasy OR "3,4-Methylenedioxymethamphetamine" OR "2c-e" OR "4-Bromo-2,5-dimethoxyamphetamine" OR "2c-b" OR Psilocin OR "2,5-Dimethoxy-4-methylamphetamine" OR nbome OR iboga OR bufotenin OR Ayahuasca OR ibogaine OR N,N-Dimethyltryptamine OR Psilocybin OR Mescaline OR Peyote OR DMT OR lysergic OR LSD OR Hallucinogen OR Psychedelic) AND (amnes* OR Neuropsychological OR Intelligence OR Attention OR Cognitive OR Learning OR Memory OR Neurocognitive OR Neuropsychiatric OR Psychological OR Psychometric OR Recall OR recognition OR "executive function" OR Aphasia OR Agnosia OR apraxia OR "Wisconsin Card" OR "Boston Naming" OR "Rey osterrieth" OR MMSE OR Stroop OR "Digit span" OR "trail making" OR "Word list" OR fluency) |
| --- |

Supplemental Table S2: Included studies with psychedelics investigating the major domains, memory, executive functioning and attention.

|  | Study Design | | | Sample characteristics | Assessed neuropsychological domains | | | Rating of bias/quality |
| --- | --- | --- | --- | --- | --- | --- | --- | --- |
| Study | Conditions | Dosage | Study type | N (female), Age in years | MEM | EXE | ATT |  |
| Abramson, Jarvik, Hirsch (1955a) | LSD  No Substance | LSD: 50, 100 µg | Sequential | Experimental: 12 (6), Median age: 27, Range: 21-33 | - | - | Manual simple reaction time, manual complex reaction time, verbal reaction time* | NHLBI tool: Poor |
| Aronson et al. (1962) | LSD  Placebo | LSD: 100 µg | Parallel | Experimental: 20 (0), -  Control: 20 (0), - | Word Learning Task | - | - | Cochrane RoB: High risk |
| Barendregt (1960) | LSD  No substance | LSD: 100-150 µg | Sequential | Experimental: 21 (3), -, Range: 25- 50 years | Figure reconstruction test* | - | - | NHLBI tool: Poor |
| Barret et al. (2018) | Psilocybin  DXM  Placebo | Psilocybin: 10, 20, 30 mg/ 70 kg  DXM: 400 mg/ 70 kg | Cross-over | Experimental: 20 (11), Range: 22 – 43, Mean: 28.5 | Self-designed encoding task*; N-back task* | Stroop; DSST* | - | Cochrane RoB: High risk |
| Brengelman (1958) | LSD  Placebo | LSD: 60 µg | Sequential | Experimental: - (-), -, Range: 25-31 | Figure reconstruction test* | - | - | NHLBI tool: Poor |
| Bouso et al. (2013) | Ayahuasca | Ayahuasca: 100ml | Sequential | Experimental: 24 (12), Mean: 45.8 | Sternberg task* | Stroop+; Tower of London | - | NHLBI tool: Poor |
| Carter et al. (2005) | Psilocybin  Ketanserin  Placebo | Psilocybin: 215 µg/kg  Ketanserin: 50mg | Cross-over | Experimental: 8 (3), Range: 21 – 31, Mean: 27 | Block tapping | - | Object tracking*** | Cochrane RoB: High risk |
| Cohen et al. (1962) | Sernyl  Amobarbital + Amphetamine  LSD  Schizophrenic Patients | Sernyl: 0.1 mg/KG  Amobarbital + Amphetamine: 500 mg + 15 mg  LSD: 1 µg/kg | Parallel | Experimental (Sernyl): 10 (-), -  Experimental (Amobarbital + Amphetamine): 5 (-), -  Experimental (LSD): 10 (-), -  Schizophrenic Controls: 10 (-), - | - | Serial sevens | - | Cochrane RoB: High risk |
| Daumann et al. (2008) | DMT  Ketamine  Placebo | DMT: 0.17 mg/kg  Ketamine: 0.115 mg/kg | Cross-over | Experimental: 14 (6), Range: 26 – 42, Mean: 32.1 | - | - | COVAT* | Cochrane RoB: High risk |
| Dittrich (1971) | Mescaline  Placebo | Mescaline: 100 mg | Cross-over | Experimental: 1 (0), Age: 29 | - | - | d2 test | Cochrane RoB: High risk |
| Duke & Keeler (1968) | Psilocybin  Amphetamine  Placebo | Psilocybin: 0.2 mg/kg  Amphetamine: 30 mg | Cross-over | Experimental: 8 (0), - | - | TMT – B* | TMT – A* | Cochrane RoB: High risk |
| Gastaut et al. (1953) | LSD  No substance | LSD: 0.5 µg /kg | Sequential | Experimental: 12 (0), - , Range: 25-50 | - | - | Lahy attention test | NHLBI tool: Poor |
| Goldberger (1966) | LSD  Placebo  Isolation | LSD: 100 µg | Parallel | Experimental (LSD): 14 (-), -  Experimental (Isolation): 14 (-), -  Control: 14 (-), - | Digit Span | Numbers*, Rhymes*, Serial Sevens* | - | Cochrane RoB: High risk |
| Gouzoulis-Mayfrank et al. (2002) | Psilocybin  MDE  Amphetamine  Placebo | Psilocybin: 0.2 mg/kg, max. 15 mg  MDE: 2 mg/kg, max. 140 mg  Amphetamine: 0.2 mg/kg, max. 17.5 mg | Parallel | Psilocybin: 8 (-), -  MDE: 8 (-), -  Amphetamine: 8 (-), -  Placebo: 8 (-), - | - | - | COVAT* | Cochrane RoB: High risk |
| Gouzoulis-Mayfrank et al. (2006) | DMT  Ketamine | DMT: 0.215, 0.32 mg/kg  Ketamine: 0.16, 0.215 mg/kg | Sequential | Experimental: 15 (6), Range: 28 – 35, Mean: 38 | - | - | COVAT* | NHLBI tool: Poor |
| Harman et al. (1966) | Mescaline  No substance | Mescaline: 200 mg | Sequential | Experimental: 27 (0), -, - | - | Purdue creativity test+ | - | NHLBI tool: Poor |
| Hasler et al. (2004) | Psilocybin  Placebo | Psilocybin: 45, 115, 215, 315 µg/kg | Cross-over | Experimental: 8 (4), Range: 22 – 44, Mean: 29.5 | - | - | FAIR* | Cochrane RoB: High risk |
| Heekern et al. (2007) | DMT  Ketamine  No substance | DMT: 0.15-0.3 mg/Kg initial dose + 0.01125 – 0.02mg/kg*min continuous  Ketamine: 0.1-0.2 mg/kg initial dose + 0.0066 – 0.015 mg/kg*min continuous | Cross-over | Experimental: 15 (6), Mean age: 38.0, Range: 28-53 | - | - | Startle reaction | Cochrane RoB: High risk |
| Heekeren et al. (2008) | DMT  Ketamine  No substance | DMT: 0.215, 0.32 mg/kg  Ketamine: 0.11, 0.215 mg/kg | Cross-over | Experimental: 15 (6), Range: 28 – 53, Mean: 38 | - | - | AX – CPT | Cochrane RoB: High risk |
| Heim et al. (1968) | LSA  Placebo  Psilocybin | LSA: -  Psilocybin: - | Parallel | Experimental (LSA): 10 (-), -  Experimental (psilocybin): 24 (-), -  Control: ­100 (-), - | LSA: Word repetition forward, backwards*; Psilocybin: Word repetition | - | - | Cochrane RoB: High risk |
| Jarvik et al. (1955) | LSD  Placebo | LSD: 50, 100 µg | Sequential | Experimental: 12 (6), median age: 27 | letter recognition*, nonsense syllable recognition, object recall*, word opposite recall*, nonsense syllable recall*, sentence recall*, paired associate recall*, digit span, unrelated word recall | - | - | NHLBI tool: Fair |
| Kanen et al. (2022) | LSD  Placebo | LSD: 75 µg | Cross-over | Experimental: 19 (4), Mean age: 30.6 | - | Probabilistic reversal learning task | - | Cochrane RoB: High risk |
| Kometer et al. (2012) | Psilocybin  Placebo | Psilocybin: 215 µg/kg | Cross-over | Experimental: 17 (6), Mean age: 26, - | - | Go/No-Go Task* | - | Cochrane RoB: High risk |
| Kornetsky et al. (1956) | Meperidine  Secobarbital  Chlorpromazine  LSD  Placebo | Meperidine: 50, 100 mg  Secobarbital: 100, 200 mg  Chlorpromazine: 200, 400 mg  LSD: 50, 100 µg | Cross-over | Experimental: 10 (4), Mean: 20.05 | - | Digit-Symbol coding*, Speed of Addition* | Copying Numbers | Cochrane RoB: High risk |
| Kuypers et al. (2016) | Ayahuasca | Ayahuasca: - | Sequential | Experimental: 26 (17), Mean: 44.7 | - | PLMT, *PCT* creativity, creativity/fluency+ | - | NHLBI tool: Poor |
| Lienert (1966) | LSD  No substance | LSD: 1.25 µg/kg | Sequential | Experimental: 65 (-), Mean: 24.3 | *IST* Concept memory | *IST* number series, spatial relations, cube rotation | - | NHLBI tool: Poor |
| Mallaroni et al. (2023) | 2C-B  Psilocybin  Placebo | 2C-B: 20mg  Psilocybin: 15mg  Placebo | Cross-over | Experimental: 22 (11), Mean age: 25, range: 19-35 years | Spatial memory task* | DSST*, TOL* | Psychomotor vigilance task* | Cochrane RoB: High risk |
| Mason et al. (2021) | Psilocybin  Placebo | Psilocybin: 0.17 mg/kg | Parallel | Experimental: 30 (12), mean age: 22.7 Control: 30 (13), mean age: 23.2 | - | *PCT* fluency*, originality*, ratio, convergent*; *AUT* fluency*, originality, ratio, novel | - | Cochrane RoB: High risk |
| Netz, Jonsson, Bergqvist (1963) | LSD  Placebo | LSD: 75-100 µg | Cross-over | Experimental: 8 (3), Mean age: 22.5, Range: 19-27 | - | - | Simple* and disjunctive* reaction time | Cochrane RoB: High risk |
| Nikolic et al. (2023) | Psilocybin  Placebo | Psilocybin: 0.26 mg/kg | Cross-over | Experimental: 20 (10), Mean age: 36, Range: 28-53 | Groton maze learning task, Rey auditory verbal learning task, paired associative learning test | - | - | Cochrane RoB: High risk |
| Ostfeld (1962) | LSD  JB-318  No substance | LSD: 75-100 µg  JB-318: 7.5 – 12.5 mg | Sequential | Experimental: 10 (-),-,- | - | Stroop* | - | NHLBI tool: Poor |
| Pokorny et al. (2019) | LSD  Ketanserin  Placebo | LSD: 100 µg  Ketanserin: 40 mg | Cross-over | Experimental: 25 (6), Mean: 25.24 | SWM* | WCST *; Cambridge Gambling Task | - | Cochrane RoB: High risk |
| Primac et al. (1956) | Meperidine  Secobarbital  Chlorpromazine  LSD  Placebo | Meperidine: 50, 100 mg  Secobarbital: 100, 200 mg  Chlorpromazine: 200, 400 mg  LSD: 50, 100 µg | Cross-over | Experimental: 10 (4), Mean: 20.05 | - | WCST | CPT | Cochrane RoB: High risk |
| Quednow et al. (2012) | Psilocybin  Ketanserin  Placebo | Psilocybin: 260 µg/kg  Ketanserin: 40mg | Cross-over | Experimental: 16 (3), Range: 24 – 39, Mean: 29.7 | - | Stroop* | - | Cochrane RoB: High risk |
| Ramaekers et al. (2023) | Ayahuasca  No substance | Mean ayahuasca dosage: 24ml | Sequential | Experimental: 14 (10), Mean age: 55.2 | - | CFAT | - | NHLBI tool: Poor |
| Resnick, Krus, Raskin (1964) | LSD  No substance | LSD: 75 µg | Sequential | Experimental: 4 (-), -, - | - | Stroop* |  | NHLBI tool: Poor |
| Resnick, Krus, Raskin (1965) | LSD  No substance | LSD: 75 µg | Sequential | Experimental: 3 (-), -, - | - | Stroop* | - | NHLBI tool: Poor |
| Rosenbaum et al. (1959) | Same as Cohen et al. (1962) | Same as Cohen et al. (1962) | Same as Cohen et al. (1962) | Same as Cohen et al. (1962) | - | - | Reaction time | Cochrane RoB: High risk |
| Rynearson, Wilson, Bickford (1968) | Psilocybin  No substance | Psilocybin: 10 mg | Cross-over | Experimental: 22 (3), -, - | - | - | Reaction time* | Cochrane RoB: High risk |
| Safer (1970) | LSD  No substance | LSD: 1.5 – 2 µg/kg | Sequential | Experimental: 10 (0), -, - | Digit recall | - | Vigilance task | NHLBI tool: Poor |
| Schmidt et al. (2017) | LSD  Placebo | LSD: 100 µg | Cross-over | Experimental: 18 (9), mean age: 31 | - | Go/No-Go task* | - | Cochrane RoB: High risk |
| Silverstein & Klee (1958) | LSD  Placebo | LSD: 72 µg | Cross-over | Experimental: 16 (0), - | Wechsler Memory scale* | - | - | Cochrane RoB: High risk |
| Silverstein & Klee (1960) | LSD  Placebo | LSD: 2 µg/kg | Cross-over | Experimental: 24 (0), - | Digit span* | - | - | Cochrane RoB: High risk |
| Sjoerdsma, Kornetsky, Evarts (1956) | LSD  No substance | LSD: 40-80 µg | Sequential | Experimental: 2 (1), - ,- | - | - | Digit copying* | NHLBI tool: Poor |
| Sloane & Doust (1954) | LSD | LSD: 40, 120 µg | Cross-over | Healthy: 14 (7), mean age: 28  Schizophrenic: 7 (0), mean age: 28  Depressed: 12 (3), mean age: 41 | Digit span; Wittenborn remembered numbers; Wechsler Memory scale; | - | Estimating lengths of time, Serial sevens* | Cochrane RoB: High risk |
| Snyder et al. (1968) | DOM  Placebo | DOM: 2.7, 3.3 mg | Parallel | Experimental (2.7 mg): 2 (-), -  Experimental (3.3 mg): 4 (-), -  Control: 6 (-), - | Serial Learning Task+ | - | - | Cochrane RoB: High risk |
| Spitzer et al. (1996) | Psilocybin Placebo | Psilocybin: 0.2 mg/kg | Cross-over | Experimental: 8 (0), Mean age: 39.4 | - | - | Reaction time* | Cochrane RoB: High risk |
| Thatcher, Wiederholt, Fischer (1971) | Psilocybin  No substance | Psilocybin: 160 µg/kg | Sequential | Experimental: - (-), Median age: 23, - | - | - | Reaction time* | NHLBI tool: Fair |
| Umbricht et al. (2003) | Psilocybin  Placebo | Psilocybin: 0.28 mg/kg | Cross-over | Experimental: 18 (8), Mean: 25.1 | - | - | AX-CPT * | Cochrane RoB: High risk |
| Vollenweider et al. (1998) | Psilocybin  Ketanserin  Haloperidol  Risperidone  Placebo | Psilocybin: 0.25 mg/kg  Ketanserin: 20, 40 mg  Haloperidol: 0.021 mg  Risperidone: 0.5, 1 mg | Parallel | Placebo/Ketanserin + Placebo/Psilocybin: 5 (-), -  Placebo/Haloperidol + Placebo/Psilocybin: 5 (-), -  Placebo/Risperidone + Placebo/Psilocybin: 5 (-), - | Delayed response task* | - | - | Cochrane RoB: High risk |
| Vollenweider et al. (2007) | Psilocybin  Placebo | Psilocybin: 115, 215, 315 µg/kg | Cross-over | Experimental: 16 (9), Range: 21 – 32, Mean: 26.4 | - | - | FAIR* | Cochrane RoB: High risk |
| Wapner & Krus (1960) | LSD  Placebo | LSD: 75 µg | Cross-over | Experimental: 24 (12), Mean: 30, Range: 20-57 | - | Stroop* | - | Cochrane RoB: High risk |
| Weingartner et al. (1971) | DOM  Placebo | DOM: 2.7, 3.3 mg | Parallel | Experimental (2.7 or 3.3mg): 6 (0), -  Control: 6 (0), - | Serial Learning Task | - | - | Cochrane RoB: High risk |
| Wießner et al. (2022) | LSD  Placebo | LSD: 50 µg | Cross-overs | Experimental: 24 (8), mean age: 35, range: 25-61 | - | PMT+; AUT; PCT*; MET; FIG+ | - | Cochrane RoB: High risk |
| Wittmann et al. (2007) | Psilocybin  Placebo | Psilocybin: 115, 250 µg/kg | Cross-over | Experimental: 12 (6), Mean: 26.8 | Block tapping | - | - | Cochrane RoB: High risk |
| Zegans, Pollards, Brown (1967) | LSD  Placebo | LSD: 0.5 µg/kg | Parallel | LSD: 19 (-), -, -  Placebo: 11 (-), -, - | - | Remote association, modified word association+, mosaic design* |  | Cochrane RoB: High risk |
| Summary | | | | | Impairment: 12 Tasks  Improvement: 1 Task  No Effect: 15 Tasks | Impairment: 19 Tasks  Improvement:6 Tasks  No Effect: 15 Tasks | Impairment: 17 Tasks  Improvement: 0 Tasks  No Effect: 11 Tasks |  |
| *Note:* *p < 0.05 in favor of control condition; + p < 0.05 in favor of drug condition; LSD, lysergic acid diethylamide; LSA, lysergic acid amide; N, sample size; MEM, memory; EXE, executive functions; ATT, attention; DSS, Digit Symbol Substitution Task; Rey-OFC, Rey-Osterrieth Complex Figure task; IST, Intelligenz-Struktur Test; SWM, Spatial Working Memory; WCST, Wisconsin Card Sorting Task; CPT, Continuous Performance Task; PMT, Pattern Meaning Task; AUT, Alternate Uses Task; PCT, Picture Concept Task; MET, creative metaphors task; FIG, figural creativity task; DOM, dimethoxy methylamphetamine; TAT, Thematic Apperception Test; DMT, dimethyltryptamine; MDE, methylenedioxyethylamphetamine; DXM, dextromethorphan; COVAT, Covert Orienting of Attention Test; TMT; Trail Making Test; FAIR, Frankfurt Attention Inventory; PLMT, Pattern/Line Meaning task; AX-CPT, AX – Continuous Performance task; CFAT, Chain-Free Association Task. | | | | | | | | |

Supplemental Table S3. MDMA Studies investigating the major domains, memory, executive functioning and attention

|  | Study Design | | | Sample characteristics | Assessed neuropsychological domains | | | Rating of bias/quality |
| --- | --- | --- | --- | --- | --- | --- | --- | --- |
| Study | Conditions | Dosage | Study type | N (female), Age in years | MEM | EXE | ATT |  |
| Bosker et al. (2010) | MDMA  Placebo | 25, 50, 100 mg | Cross-over | Experimental: 16 (8), Mean: 22.0 | Rapid information processing | Stop-Signal | Divided Attention task; psychomotor vigilance task | Cochrane RoB: High risk |
| Curran & Travill (1997) | MDMA;  Alcohol | n/a | Parallel | Experimental: 12 (4), -  Alcohol: 12 (4), - | Prose recall; serial sevens* | - | - | Cochrane RoB: High risk |
| De Sousa Fernandes Perna et al. (2014) | Placebo-Placebo;  Memantine – Placebo;  Placebo-MDMA;  Memantine-MDMA | MDMA 75 mg;  Memantine 20 mg | Cross-over | Experimental: 15 (4), Mean: 22.2 | Visual Verbal Learning Task*; PMT; Sternberg memory task+; Abstract Visual Pattern Learning Task* | - | Critical Tracking Task; Divided Attention Task | Cochrane RoB: High risk |
| Doss et al. (2017) | MDMA;  Placebo | MDMA 1mg/KG | Parallel | Retrieval: 10 (10), Mean: 22.45  Placebo: 10 (10), Mean: 23.85  Encoding: 10 (10), Mean: 24.9 | Cued recollection; Picture recognition | - | - | Cochrane RoB: High risk |
| Downing (1986) | MDMA | Various | Sequential | Experimental: 10 (-), - | Digit repetition | - | - | NHLBI tool: Poor |
| Dumont et al. (2008) | MDMA + Alcohol;  Placebo + Alcohol; MDMA + Placebo; Placebo + Placebo | MDMA 100 mg;  Alcohol BAC 0.6 promille | Cross-over | Experimental: 14 (-), Mean: 22.1 | Auditory Verbal Learning Task*; Symbol digit recall test* | Switch task | SDST* | Cochrane RoB: High risk |
| Dumont, Schoemaker et al. (2010) | MDMA + Alcohol;  Placebo + Alcohol; MDMA + Placebo; Placebo + Placebo | MDMA 100 mg;  Alcohol BAC 0.6 promille | Cross-over | Experimental: 14 (-), Mean: 22.1 | - | - | Simple  reaction time | Cochrane RoB: High risk |
| Dumont, van Hasselt et al. (2011) | MDMA + THC;  Placebo + THC; MDMA + Placebo; Placebo + Placebo | MDMA 100 mg;  THC 4+6+6 mg over 90 minutes | Cross-over | Experimental: 16 (4), Mean: 21.0 | Auditory Verbal Learning Task*; N-back Task | - | Simple  reaction time | Cochrane RoB: High risk |
| Gamma et al. (2000) | MDMA;  Placebo | MDMA 1.7 mg/Kg | Cross-over | Experimental: 16 (6), Mean: 26.0 | - | - | AX-CPT | Cochrane RoB: High risk |
| Haijen et al. (2018) | MDMA  Ketanserin  Placebo | MDMA: 75 mg  Ketanserin: 40 mg | Cross-over | Experimental: 20 (8), Mean age: 21.2 | Word learning task* | - | - | Cochrane RoB: High risk |
| Hasler et al. (2009) | MDMA  Pindolol  Placebo | MDMA: 1.6 mg/kg  Pindolol: 20 mg | Cross-over | Experimental: 15 (0), Mean age: 24.3, range: 20-36 | RVP*, PAL* | - | ID/ED | Cochrane RoB: High risk |
| Hoshi et al. (2006) | MDMA  No substance | MDMA: / | Parallel | Experimental: 19 (8), - | Sentence recognition | - | - | Cochrane RoB: High risk |
| Hysek et al. (2012) | MDMA  Placebo | MDMA: 125 mg | Cross-over | Experimental: 48 (24), mean age: 26, Range: 18-44 | - | - | Choice reaction time task | Cochrane RoB: High risk |
| Kirkpatrick et al. (2012) | MDMA  Methamphetamine  Placebo | MDMA: 100 mg  Methamphetamine: 20 mg | Cross-over | Experimental: 11 (2), Mean age: 29.3 | Digit recall, Repeated acquisition task | DSST | Divided attention task, rapid information task | Cochrane RoB: High risk |
| Kloft et al. (2022) | MDMA;  Placebo | MDMA 75 mg | Cross-over | Experimental: 61 (28), Mean: 23.0 | DRM* | - | - | Cochrane RoB: High risk |
| Kuypers & Ramaekers (2005) | MDMA; Methylphenidate;  Placebo | MDMA 75 mg; Methylphenidate 20 mg | Cross-over | Experimental: 18 (9), Mean: 26.22 | Verbal Word Learning Task*; Syntactic Reasoning Task | DSST | - | Cochrane RoB: High risk |
| Kuypers & Ramaekers (2006) | MDMA; Methylphenidate;  Placebo | MDMA 75 mg; Methylphenidate 20 mg | Cross-over | Experimental: 18 (9), Mean: 26.22 | Spatial Memory task* | - | - | Cochrane RoB: High risk |
| Kuypers, Samyn, Ramaekers (2006) | MDMA  Placebo | MDMA: 75, 100 mg | Cross-over | Experimental: 18 (9), Mean age: 26.6 | - | - | Object Movement estimation under divided attention | Cochrane RoB: High risk |
| Kuypers et al. (2007) | MDMA;  Placebo | MDMA 75 + 50 mg | Cross-over | Experimental: 14 (7), Mean: 22.93 | - | Stop-signal; Discounting task | Divided attention task*; Mackworth Clock task | Cochrane RoB: High risk |
| Kuypers, Wingen & Ramaekers (2008) | MDMA;  Placebo | MDMA 75 + 50 mg | Cross-over | Experimental: 14 (7), Mean: 22.93 | Spatial Memory task*; Sternberg memory scanning task*; Star Counting task* | - | - | Cochrane RoB: High risk |
| Kuypers et al. (2011) | MDMA;  Placebo | MDMA 75 mg | Cross-over | Experimental: 14 (3), Mean: 23.43 | Word learning task* | - | - | Cochrane RoB: High risk |
| Kuypers et al. (2013) | Placebo + MDMA;  Metyrapone + MDMA;  Placebo + Placebo;  Metyrapone + Placebo | MDMA 75 mg;  Metyrapone 750 mg | Cross-over | Experimental: 14 (3), Mean: 23.43 | Word learning task*; continuous recognition memory task*; spatial memory task*; Sternberg memory task, prospective memory task* | - | - | Cochrane RoB: High risk |
| Kuypers et al. (2014) | Pindolol + MDMA + Placebo;  Placebo + MDMA + Placebo; Placebo + Placebo + Oxytocin; Placebo + Placebo + Placebo | MDMA 75 mg;  Pindolol 20 mg; Oxytocin 40 IU + 2x8 IU | Cross-over | Experimental: 20 (8), Mean: 21.6 | Word learning task* | - | - | Cochrane RoB: High risk |
| Lamers et al. (2003) | MDMA  Alcohol  Placebo | MDMA 75 mg;  Alcohol 0.5 g/kg ethanol | Cross-over | Experimental: 12 (4), Mean age: 23.5, Range: 21-30 | - | Word fluency, TOL | Divided attention task*, movement under divided attention, visual search | Cochrane RoB: High risk |
| Parrot & Lasky (1998) | MDMA;  No Drug | MDMA Various quantities in form of self-administered ecstasy | Sequential | Experienced MDMA User: 15 (-), Mean: 21.4  Novice MDMA User: 15 (-); Mean: 22.8  Controls: 15 (-); Mean: 21.3 | Auditory word recall* | - | - | NHLBI tool: Poor |
| Ramaekers & Kuypers (2006) | MDMA;  Alcohol;  Placebo | MDMA 75, 100 mg  Alcohol BAC 0.6 promille | Cross-over | Experimental: 18 (9), Range: 20-37 | - | Stop-Signal*; Go/no-Go task | - | Cochrane RoB: High risk |
| Ramaekers et al. (2009) | MDMA  Placebo | MDMA 75 mg | Cross-over | Experimental: 12 (3), Mean age: 23.3, Range: 19-29 | PMT* | - | - | Cochrane RoB: High risk |
| Schmidt et al. (2017) | MDMA;  Placebo;  Methylphenidate;  Modafinil | MDMA 125 mg;  Methylphenidate 60 mg;  Modafinil 600 mg | Cross-over | Experimental: 18 (9), Range: 20-37 | - | Go/no-Go task | - | Cochrane RoB: High risk |
| Spronk et al. (2014) | MDMA + Alcohol;  Placebo + Alcohol; MDMA + Placebo; Placebo + Placebo | MDMA 100 mg;  Alcohol BAC 0.6 promille | Cross-over | Experimental: 14 (-), Mean: 22.1 | - | Flanker task | - | Cochrane RoB: High risk |
| Stough et al. (2012) | MDMA;  d-Methamphetamine;  Placebo | MDMA 100 mg;  d-methamphetamine 0.42 mg/Kg | Cross-over | Experimental: 61 (33), Mean: 25.45 | Spatial working memory; Numeric working memory | TMT-B* | Simple reaction time; Digit vigilance; Choice reaction time; TMT-A* | Cochrane RoB: High risk |
| Van Wel et al. (2011) | MDMA  Placebo | MDMA 75 mg | Cross-over | Experimental: 17 (8), Mean age: 22.76, Range: 19-27 | Word learning task*, Spatial memory task; PMT | - | - | Cochrane RoB: High risk |
| Van Wel et al. (2012) | Placebo + Placebo; Pindolol + Placebo; Ketanserin + Placebo; Placebo + MDMA; Pindolol + MDMA; Ketanserin + MDMA | MDMA 75 mg;  Pindolol 20 mg;  Ketanserin 50 mg | Cross-over | Experimental: 17 (9), Mean: 22.76 | - | Matching familiar figures; Stop-Signal*; Cue-reversal | - | Cochrane RoB: High risk |
| Vollenweider et al. (1998) | MDMA;  Placebo | MDMA 1.7 mg/Kg | Cross-over | Experimental: 13 (3), Mean: 29 | - | Stroop | - | Cochrane RoB: High risk |
| Summary | | | | | Impairment: 23 Tasks  Improvement: 1 Task  No Effect: 13 Tasks | Impairment: 4 Tasks  Improvement: 0 Task  No Effect: 14 Tasks | Impairment: 4 Tasks  Improvement: 0 Task  No Effect: 17 Tasks |  |

*Note*: * p < 0.05 in favor of control condition; + p < 0.05 in favor of drug condition; MDMA, methylenedioxymethamphetamine; BAC, blood alcohol content; THC, tetrahydrocannabinol; N, sample size; MEM, memory; EXE, executive functions; ATT, attention; AX-CPT, AX continuous performance task; DRM, Deese/Roediger–McDermott word list paradigm; SDST + DSST, Digit Symbol Substitution Task; TMT, Trail-Making Test; PMT, prospective memory task; RVP, Rapid visual processing; PAL, paired associates learning; ID/ED, Intradimensional attentional shift

Supplemental Table S4. Microdosing studies investigating the major domains, memory, executive functioning and attention

|  | Study Design | | | **Sample characteristics** | Assessed neuropsychological domains | | | Rating of bias/quality |
| --- | --- | --- | --- | --- | --- | --- | --- | --- |
| Study | Conditions | Dosage | Study type | **N (female), Age in years** | MEM | EXE | ATT |  |
| Bershad et al. (2019) | LSD  Placebo | LSD: 6.5, 13, 26 µg | Cross-over | Experimental: 20 (12), Mean: 25 | n-back Task | DSST; RAT | - | Cochrane RoB: High risk |
| Cavanna et al. (2022) | Psilocybin  Placebo | Psilocybe Cubesins: 0.5 g | Cross-over | Experimental: 34 (11), Mean: 31.2 | - | RAT; AUT; WK; TMT-B; Go/no-Go; Stroop* | TMT-A; Attentional Blink | Cochrane RoB: High risk |
| Fallon et al. (2021) | LSD  Placebo | LSD: 5, 10, 20 µg | Parallel | Experimental (5 µg): 12 (6), Mean: 63.5  Experimental (10 µg): 12 (6), Mean: 63.17  Experimental (20 µg): 12 (3), Mean: 61.58  Control: 12 (6), Mean: 63.42 | Ignore/Update Task | - | - | Cochrane RoB: High risk |
| Family et al. (2019) | LSD  Placebo | LSD: 5, 10, 20 µg | Parallel | Same as Fallon et al. (2021) | PAL, SWM | - | RVP; RTI | Cochrane RoB: High risk |
| Hutten et al. (2020) | LSD  Placebo | LSD: 5, 10, 20 µg | Cross-over | Experimental: 24 (12), mean age: 22.8 | - | DSST*; cognitive control task | Psychomotor vigilance task | Cochrane RoB: High risk |
| Prochazkova et al. (2018) | Psilocybin truffles | Truffles: 0.22, 0.33, 0.44 g | Sequential | Experimental: 27 (9), Mean: 31.5 | - | *PCT* convergent+; *AUT* fluency, flexibility and originality+, | - | NHLBI tool: Poor |
| de Wit et al. (2022) | LSD  Placebo | LSD: 13, 26 µg | Parallel | Experimental (13 µg): 19 (6), Mean: 26.6  Experimental (26 µg): 19 (6), Mean: 25.9  Control: 18 (7), Mean: 24.4 | n-back Task | DSST | - | Cochrane RoB: High risk |
| Yanakieva et al. (2019) | LSD  Placebo | LSD: 5, 10, 20 µg | Parallel | Same as Family et al. (2019) | - | - | Temporal reproduction task*** | Cochrane RoB: High risk |
| Summary | | | | | Impairment: 0 Tasks  Improvement: 0 Task  No Effect: 5 Tasks | Impairment: 2 Tasks  Improvement: 2 Task  No Effect: 11 Tasks | Impairment: 1 Tasks  Improvement: 0 Task  No Effect: 5 Tasks |  |
| *Notes::* * p < 0.05 in favor of control condition; + p < 0.05 in favor of drug condition; LSD, Lysergic Acid Diethylamide: N, sample size; MEM, memory; EXE, executive functions; ATT, attention; DSST, Digit Symbol Substitution Task; RAT, Remote Association Task; PCT, Picture Concept Task; AUT, Alternative Use Task; PAL, Paired Associates Learning; CANTAB, Cambridge Neuropsychological Test Automated Battery; SWM, Spatial Working Memory Task; TMT, Trail-Making Test; RVP, Rapid visual information processing; RTI, reaction time; WK, Wallach–Kogan Test. | | | | | | | | |

Supplemental Table S5. Subacute studies investigating the major domains, memory, executive functioning and attention

|  | Study Design | | | **Sample characteristics** | Assessed neuropsychological domains | | | Rating of bias/quality |
| --- | --- | --- | --- | --- | --- | --- | --- | --- |
| Study | Conditions | Dosage | Study type | **N (female), Age in years** | MEM | EXE | ATT |  |
| Kirkpatrick et al. (2012) | MDMA  Methamphetamine  Placebo | MDMA: 100 mg  Methamphetamine: 20 mg | Cross-over | Experimental: 11 (2), Mean age: 29.3 | Digit recall, Repeated acquisition task | DSST | Divided attention task, rapid information task | Cochrane RoB: High risk |
| Pirona & Morgan (2009) | MDMA  No substance | MDMA: - | Parallel | Experimental: 16 (6), Mean age: 22.81  Control: 16 (7), 22.62 | RBMT | SSS, MFF | - | Cochrane RoB: High risk |
| Kiraga et al. (2022) | Ayahuasca | Ayahuasca: 57.44 mg DMT | Sequential | Experimental: 64 (27), mean age: 39 | - | *PCT* convergent, divergent* | - | NHLBI tool: Poor |
| Mason et al. (2019) | Psilocybin | Psilocybin containing truffles: 34.2g | Sequential | Experimental: 55 (26), mean age: 34.8 | - | *PCT* convergent, divergent+ |  | NHLBI tool: Poor |
| Murphy-Beiner & Soar (2020) | Ayahuasca | Ayahuasca: - | Sequential | Experimental: 48 (26), Mean: 38.5 | - | WCST+; Stroop | - | NHLBI tool: Poor |
| Ornelas et al. (2022) | LSD  Placebo | LSD: 50 µg | Cross-over | Experimental: 24 (8), mean age: 35, range: 25-61 | 2D object-location task+ | - | - | Cochrane RoB: High risk |
| Reckweg et al. (2021) | 5-MeO-DMT | 5-MeO-DMT: 2mg, 6mg, 12mh, 18mg | Parallel | Experimental: 22 (9), Mean age: 29, range: 18-42 | PMT | DSST | PVT | Cochrane RoB: High risk |
| Uthaug et al. (2018) | Ayahuasca | Ayahuasa: 189.4 – 915.4 mg DMT | Sequential | Experimental: 57 (36), - | - | *PCT* convergent+, divergent | - | NHLBI tool: Poor |
| Uthaug et al. (2019) | 5-MeO-DMT | 5-MeO-DMT: 203.6 – 307.3 mg/g | Sequential | Experimental: 42 (17), Mean: 38.0 | - | *PCT* convergent+, divergent | - | NHLBI tool: Poor |
| Wießner et al. (2022) | LSD  Placebo | LSD: 50 µg | Cross-over | Same as Ornelas et al. (2022) | ROCF+; OLMT+; RAVLT | WCST*; TMT-B; Stroop | TMT-A | Cochrane RoB: High risk |
| Summary | | | | | Impairment: 0 Tasks  Improvement: 3 Task  No Effect: 5 Tasks | Impairment: 2 Tasks  Improvement: 4 Task  No Effect: 11 Tasks | Impairment: 0 Tasks  Improvement: 0 Task  No Effect: 4 Tasks |  |
| *Notes::* * p < 0.05 in favor of control condition; + p < 0.05 in favor of drug condition; DMT, dimethyltryptamine; 5-MeO-DMT, 5-methoxy-dimethyltryptamine; N, sample size; MEM, memory; EXE, executive functions; ATT, attention; PCT, Picture Concept Task; WCST, Wisconsin Card Sorting Test; TMT, Trail-Making Test; ROCF, Rey-Osterrieth Comple Figure task; RAVLT, Rey Auditory Verbal Learning Test; OLMT, 2D Object-Location Memory Task; RBM, Riveheard behavioral memory test; SSS, serial seven subtractions, MFF, matching familiar figures | | | | | | | | |

Supplemental Table S6. Psychedelic studies investigating visuo-spatial abilities, general intelligence, verbal abilities and motor function.

|  | Study Design | | | Sample characteristics | Assessed neuropsychological domains | | | | Qualtiy/RoB |
| --- | --- | --- | --- | --- | --- | --- | --- | --- | --- |
| Study | Conditions | Dosage | Study Type | N (female), Age in years | VSA | INT | VER | MOT |  |
| Abramson, Jarvik, Hirsch (1955b) | LSD  No Substance | LSD: 50, 100 µg | Sequential | Same as Abramson, Jarvik, Hirsch (1955a) | - | - | - | Pursuit rotor test*, steadiness test* | NHLBI tool: Poor |
| Abramson, Jarvik, Hirsch, Ewald (1955) | LSD  No Substance | LSD: 50, 100 µg | Sequential | Same as Abramson, Jarvik, Hirsch (1955a) | Thurstone Hand Test*, Minnesota Paper Form Test | - | - | - | NHLBI tool: Poor |
| Abramson et al. (1955) | LSD  No Substance | LSD: 50, 100 µg | Sequential | Experimental: 26 (13), Mean age: 27, - | Bender-Gestalt* | - | - | - | NHLBI tool: Poor |
| Barret et al. (2018) | Psilocybin  DXM  Placebo | Psilocybin: 10, 20, 30 mg/ 70 kg  DXM: 400 mg/ 70 kg | Cross-over | Experimental: 20 (11), Range: 22 – 43, Mean: 28.5 | Penn-line orientation test | - | - | Motor-praxis* | Cochrane RoB: High risk |
| Brengelman (1958) | LSD  Placebo | LSD: 60 µg | Sequential | Experimental: - (-), -, Range: 25-31 | - | - | - | Figure reconstruction test* | NHLBI tool: Poor |
| Family et al. (2016) | LSD  Placebo | LSD: 40-80 µg | Sequential | Experimental: 10 (1), Mean age: 34.2, Range: 26-47 | - | - | Object naming | - | NHLBI tool: Poor |
| Gastaut et al. (1953) | LSD  No substance | LSD: 0.5 µg /kg | Sequential | Experimental: 12 (0), - , Range: 25-50 | - | Cattell Culture Fair Intelligence Test* | - | - | NHLBI tool: Poor |
| Goldberger (1966) | LSD  Placebo  Isolation | LSD: 100 µg | Parallel | Experimental (LSD): 14 (-), -  Experimental (Isolation): 14 (-), -  Control: 14 (-), - | - | - | Comprehension*, Word Naming*, Simple Rhyming* | - | Cochrane RoB: High risk |
| Gonzalez, Torrens & Farré (2015) | 2C-B | 2C-B: 20mg | Cross-over | Experimental (2C-B): 20 (12), mean age: 34.65 years, range: 27-49 years | - | - | Verbal fluency | - | Cochrane RoB: High risk |
| Heim et al. (1968) | LSA  Placebo  Psilocybin | LSA: -  Psilocybin: - | Parallel | Experimental (LSA): 10 (-), -  Experimental (psilocybin): 24 (-), -  Control: ­100 (-), - | Rey-OFC* | - | - | - | Cochrane RoB: High risk |
| Hollister et al. (1961) | Psilocybin  Ditran  α-Methyltryptamine | Psilocybin: 60-209 µg/kg  Ditran: 40 – 339 µg/kg  α-Methyltryptamine: 384-810 µg/kg | Sequential | Experimental: 16 (-), -, range: 22-44 | FC* | NF* | - | - | NHLBI tool: Poor |
| Hollister (1961) | Psilocybin  No substance | Psilocybin: 60-209 µg/kg | Sequential | Experimental: 16 (-), -, range: 22-42 | FC* | NF* | - | - | NHLBI tool: Poor |
| Hollister, Magnicol & Gillespie (1969) | DOM  No substance | DOM: 2-14 mg | Sequential | Experimental: 18 (-), - | FC* | NF* | - | - | NHLBI tool: Poor |
| Landis & Clausen (1954) | LSD  Mescaline  No substance | LSD: 0.1 – 0.18 mg  Mescaline: 0.35-0.5 g | Parallel | LSD: 4 (-), -,-  Mescaline: 5 (-),-,-  No substance: 4 (-),-,- | - | - | - | Purdue pegboard*, Tapping speed* | Cochrane RoB: High risk |
| Levine et al. (1955) | LSD | LSD: 50-200 µg | Cross-overs | Experimental: 21 (12), median age: 29 | - | Wechsler-Bellevue Scale* | - | - | NHLBI tool: Poor |
| Lienert (1966) | LSD  No substance | LSD: 1.25 µg/kg | Cross-over | Experimental: 65 (-), Mean age: 24.3 | - | *IST t*otal*, general information, arithmetical reasoning | *IST* word classification, verbal analogies, verbal relations | - | NHLBI tool: Poor |
| Netz, Jonsson, Bergqvist (1953) | LSD  Placebo | LSD: 75-100 µg | Cross-over | Experimental: 8 (3), Mean age: 22.5, Range: 19-27 | Figure-ground | - | Synonyms* | - | Cochrane RoB: High risk |
| Safer (1970) | LSD  No substance | LSD: 1.5 – 2 µg/kg | Sequential | Experimental: 10 (0), -, - | - | NF* | - | Minnesota manipulation test | NHLBI tool: Poor |
| Savage (1952) | LSD | LSD: 20 µg | Cross-over | Experimental: 5 (-), - | - | Wechsler-Bellevue Scale* | - | - | NHLBI tool: Poor |
| Silverstein & Klee (1958) | LSD  Placebo | LSD: 72 µg | Cross-overs | Experimental: 16 (0), - | - | - | Gorham proverbs test* | - | Cochrane RoB: High risk |
| Snyder et al. (1968) | DOM  Placebo | DOM: 2.7, 3.3 mg | Parallel | Experimental (2.7 mg): 2 (-), -  Experimental (3.3 mg): 4 (-), -  Control: 6 (-), - | TAT* | - | - | - | Cochrane RoB: High risk |
| Weingartner et al. (1971) | DOM  Placebo | DOM: 2.7, 3.3 mg | Parallel | Experimental (2.7 or 3.3mg): 6 (0), -  Control: 6 (0), - | TAT* | - | - | - | Cochrane RoB: High risk |
| Wilson & Shagass (1964) | LSD  Ditran | LSD: 2 µg/kg  Ditran: 0.1 mg/kh | Sequential | Experimental: 11 (6), Mean age: 28.4, Range: 17-40 | Bender-Gestalt* | - | - | - | NHLBI tool: Poor |

*Note:* *p < 0.05 in favor of control condition; LSD, lysergic acid diethylamide; N, sample size; VSA, visuospatial abilities; INT, intelligence; VER, verbal functioning; MOT, motor function; Rey-OFC, Rey-Osterrieth Complex Figure task; IST, Intelligenz-Struktur Test; TAT, Thematic Apperception Test; DXM, dextromethorphan; DOM, dimethoxy methylamphetamine; FC, flexibility of closure; NF, number facility test

Supplemental Table S7: MDMA studies investigating visuo-spatial abilities, general intelligence, verbal abilities and motor function.

|  | Study Design | | | Sample characteristics | Assessed neuropsychological domains | | | | Qualtiy/RoB |
| --- | --- | --- | --- | --- | --- | --- | --- | --- | --- |
| Study | Conditions | Dosage | Comparison | N (female), Age in years | VSA | INT | VER | MOT |  |
| Agurto et al. (2020) | MDMA  Oxytocin  Placebo | MDMA: 0.75 & 1.5 mg/kg  Oxytocin: 20 IU | Cross-over | Experimental: 36 (18), Mean age: 23.6 | - | - | Free speech* | - | Cochrane RoB: High risk |
| Dumont et al. (2008) | MDMA + Alcohol;  Placebo + Alcohol; MDMA + Placebo; Placebo + Placebo | MDMA 100 mg;  Alcohol BAC 0.6 promille | Cross-over | Experimental: 14 (-), Mean: 22.1 | Tangle task | - | - | Pursuit task; Point task | Cochrane RoB: High risk |
| Dumont, van Hasselt et al. (2011) | MDMA + THC;  Placebo + THC; MDMA + Placebo; Placebo + Placebo | MDMA 100 mg;  THC 4+6+6 mg over 90 minutes | Cross-over | Experimental: 16 (4), Mean: 21.0 | - | - | - | Pursuit task | Cochrane RoB: High risk |
| Hoshi et al. (2006) | MDMA  No substance | MDMA: / | Parallel | Experimental: 19 (8), - | - | - | Sentence processing | - | Cochrane RoB: High risk |
| Kuypers & Ramaekers (2006) | MDMA; Methylphenidate;  Placebo | MDMA 75 mg; Methylphenidate 20 mg | Cross-over | Experimental: 18 (9), Mean: 26.22 | Change blindness task | - | - | - | Cochrane RoB: High risk |
| Kuypers, Samyn, Ramaekers (2006) | MDMA  Placebo | MDMA: 75, 100 mg | Cross-over | Experimental: 18 (9), Mean age: 26.6 | - | - | - | Critical tracking task | Cochrane RoB: High risk |
| Kuypers et al. (2007) | MDMA;  Placebo | MDMA 75 + 50 mg | Cross-over | Experimental: 14 (7), Mean: 22.93 | - | - | - | Critical tracking task* | Cochrane RoB: High risk |
| Kuypers et al. (2013) | Placebo + MDMA;  Metyrapone + MDMA;  Placebo + Placebo;  Metyrapone + Placebo | MDMA 75 mg;  Metyrapone 750 mg | Cross-over | Experimental: 14 (3), Mean: 23.43 | - | - | National adult reading test | - | Cochrane RoB: High risk |
| Marrone et al. (2010) | MDMA;  Methamphetamine;  Placebo | MDMA 100 mg; Methamphetamine 20, 40 mg | Cross-over | Experimental: 11 (2), Mean: 29.3 | - | - | Speech quantitiy, Verbal fluency; Filled Pauses*; Silent Pauses | - | Cochrane RoB: High risk |
| Parrot & Lasky (1998) | MDMA;  No Drug | MDMA Various quantities in form of self-administered ecstasy | Parallel | Experienced MDMA User: 15 (-), Mean: 21.4  Novice MDMA User: 15 (-); Mean: 22.8  Controls: 15 (-); Mean: 21.3 | Visual search* | - | - | - | NHLBI tool: Poor |
| Lamers et al. (2003) | MDMA  Alcohol  Placebo | MDMA 75 mg;  Alcohol 0.5 g/kg ethanol | Cross-over | Experimental: 12 (4), Mean age: 23.5, Range: 21-30 | - | - | - | Critical tracking task+, motor choice reaction time+ | Cochrane RoB: High risk |

*Note*: * p < 0.05 in favor of control condition; + p < 0.05 in favor of drug condition; MDMA, methylenedioxymethamphetamine; BAC, blood alcohol content; THC, tetrahydrocannabinol; N, sample size; VSA, visuospatial abilities; INT, intelligence; VER, verbal functioning; MOT, motor function

Supplemental Table S8: Microdosing studies investigating visuo-spatial abilities, general intelligence, verbal abilities and motor function

|  | Study Design | | | **Sample characteristics** | Assessed neuropsychological domains | | | | Qualtiy/RoB |
| --- | --- | --- | --- | --- | --- | --- | --- | --- | --- |
| Study | Conditions | Dosage | Comparison | **N (female), Age in years** | VSA | INT | VER | MOT |  |
| Prochazkova et al. (2018) | Psilocybin truffles | Truffles: 0.22, 0.33, 0.44 g | Cross-over | Experimental: 27 (9), Mean: 31.5 | - | Ravens Matrices | - | - | NHLBI tool: Poor |
| Sanz et al. (2022) | Psilocybin  Placebo | Psilocybe Cubesins: 0.5 g | Cross-over | Same as Cavanna et al. (2022) | - | - | Verbosity+, Semantic variability | - | Cochrane RoB: High risk |

*Note:* *p < 0.05 in favor of control condition; N, sample size; VSA, visuospatial abilities; INT, intelligence; VER, verbal functioning; MOT, motor function;

Supplemental Table S9: Subacute studies investigating visuo-spatial abilities, general intelligence, verbal abilities and motor function

|  | Study Design | | | **Sample characteristics** | Assessed neuropsychological domains | | | | Qualtiy/RoB |
| --- | --- | --- | --- | --- | --- | --- | --- | --- | --- |
| Study | Conditions | Dosage | Comparison | **N (female), Age in years** | VSA | INT | VER | MOT |  |
| Wießner et al. (2022) | LSD  Placebo | LSD: 50 µg | Cross-over | Experimental: 24 (8), mean age: 35, range: 25-61 | Block design | - | VFT+ | - | Cochrane RoB: High risk |
| Ornelas et al. (2022) | LSD  Placebo | LSD: 50 µg | Cross-over | Experimental: 24 (8), mean age: 35, range: 25-61 | Rey-OFC+ | - | - | - | Cochrane RoB: High risk |

*Note:* *p < 0.05 in favor of control condition; LSD, lysergic acid diethylamide; N, sample size; VSA, visuospatial abilities; INT, intelligence; VER, verbal functioning; MOT, motor function; VFT, verbal fluency task; Rey-OFC, Rey-Osterrieth Complex Figure task

*Supplemental Table 10.* Results of sensitivity analyses

| **Analysis** | **Unmodified Analysis** | **Limit to assessments of speed** | **Limit to assessments of accuracy** | **Exclude study with highest weight** | **Limit to studies with same substance (LSD)** | **Limit to studies with same substance (Psilocybin)** | **Exclude studies with low RoB rating** |
| --- | --- | --- | --- | --- | --- | --- | --- |
| MDMA - memory | *Z* = -1.06 [95% CI: -1.58; - 0.54], *p* < .001 | *Z* = -1.43 [95%CI: -12.87; 10.01], *p* = .36 | *Z* = -1.10 [95%CI: -1.65; -0.55], *p* < .001 | *Z* = -1.14 [95%CI: -1.74; -0.55], *p* = .001 | NA | NA | *Z* = -0.92 [95%CI: -1.77; -0.07], *p* = .04 |
| Psychedelics - memory | *Z* = -0.81 [95%CI: -1.78; 0.17], *p* = .10 | NA | *Z* = -0.81 [95%CI: -1.81; 0.19], *p* = .10 | *Z* = -0.83 [95%CI: -1.98; 0.32], *p* = .14 | NA | *Z* = -0.73 [95%CI: -2.78; 1.32], *p* = .27 | *Z* = -1.00 [95%CI: -2.21; 0.20], *p* = .09 |
| MDMA - executive functioning | *Z* = -0.10 [95% CI: -0.67; 0.48], *p* = .72 | NA | *Z* = -0.06 [95% CI: -0.53; 0.41], *p* = .76 | *Z* = -0.06 [95% CI: -0.66; 0.54], *p* = .82 | NA | NA | *Z* = -0.07 [95%CI: -1.04; 0.90], *p* = .86 |
| Psychedelics - executive functioning | *Z* = -1.22 [95%CI: -1.92; -0.52], *p* = .003 | *Z* = -1.54 [95%CI: -3.63; 0.55], *p* = .10 | *Z* = -0.80 [95%CI: -1.39; -0.21], *p* = .015 | *Z* = -1.48 [95%CI: -2.26; -0.71], *p* = .002 | *Z* = -0.43 [95%CI: -2.63; 1.78], *p* = .25 | *Z* = -2.11 [95%CI: -5.12; 0.91], *p* = .09 | *Z* = -1.56 [95%CI: -2.82; -0.29], *p* = .024 |
| MDMA - attention | *Z* = --0.59 [95%CI: -1.82; 0.63], *p* = .30 | *Z* = -1.11 [95%CI: -4.15; 1.92], *p* = .33 | *Z* = --0.43 [95%CI: -5.78; 4.92], *p* = .76 | *Z* = -0.83 [95%CI: -2.04; 0.37], *p* = .14 | NA | NA | *Z* = -0.60 [95%CI: -2.44; 1.24], *p* = .44 |
| Psychedelics - attention | *Z* = -2.30 [95%CI: -3.71; -0.90], *p* = .005 | *Z* = -1.99 [95%CI: -4.59; 0.61], *p* = .09 | *Z* = -3.40 [95%CI: -10.28; 3.49], *p* = .17 | *Z* = -2.92 [95%CI: -4.65; -1.18], *p* = .006 | NA | *Z* = -2.18 [95%CI: -5-49; 1.14], *p* = .13 | *Z* = -2.20 [95%CI: -4.48; 0.09], *p* = .056 |
| Microdosing - creativity | *Z* = 0.37 [95%CI: -2.51; 3.24], *p* = .64 | NA | NA | NA | NA | *Z* = 0.44 [95%CI: -12.49; 13.37], *p* = .74 | NA |

Note: MDMA, Methylenedioxymethamphetamine; LSD, Lysergic Acid Diethylamide; RoB, Risk of Bias rating; NA, analysis not available

*Supplemental Table 11.* Exclusions for the quantitative analysis.

| **Reason for exclusion** | **Excluded Studies** |
| --- | --- |
| Reported tests results verbally without statistical test results (n = 16) | ^18,20,23–25,44,53–62^ |
| Only reported p-values (n = 12) | ^15,31,37,38,63–70^ |
| Only reported the test-statistic and p-values (n = 36) | ^40,43,71,72,45,73–76,47,42,77–80,17,81–87,29,88–93,50,94–98^ |
| Only reported difference scores (n = 7) | ^16,22,26,30,35,99,100^ |
| Reported means but no standard deviations (n = 7) | ^21,27,32,101–104^ |
| Not enough studies to perform meta-analysis in investigated domain (n = 13) | ^19,33,34,36,51,52,105–111^ |

**References**

1. R Core Team. R version 4.3.2. (2023).

2. Harrer, M., Cuijpers, P., Furukawa, T. & Ebert, D. D. *Dmetar: Companion R Package For The Guide ‘Doing Meta-Analysis in R’*. (2019).

3. Chang, W. extrafont: Tools for Using Fonts. (2023).

4. Huber, N. ggbrace. (2023).

5. Wilke, C. O. & Wiernik (@bmwiernik), B. M. ggtext: Improved Text Rendering Support for ‘ggplot2’. (2022).

6. Müller, K. & Bryan, J. here: A Simpler Way to Find Your Files.

7. Viechtbauer, W. metafor: Meta-Analysis Package for R. (2023).

8. Revelle, W. psych: Procedures for Psychological, Psychometric, and Personality Research. (2023).

9. Ushey, K., Wickham, H., Software, P. & PBC. renv: Project Environments. (2023).

10. Allaire, J. J. *et al.* rmarkdown: Dynamic Documents for R. (2023).

11. Xie, Y., Allaire, J. J. & Grolemund, G. *R Markdown: The Definitive Guide*. (2023).

12. Xie, Y., Dervieux, C. & Riederer, E. *R Markdown Cookbook*. (2023).

13. Kassambara, A. rstatix: Pipe-Friendly Framework for Basic Statistical Tests. (2023).

14. Wickham, H. & RStudio. tidyverse: Easily Install and Load the ‘Tidyverse’. (2023).

15. Abramson, H. A., Jarvik, M. E., Hirsch, M. W. & Ewald, A. T. Lysergic Acid Diethylamide (LSD-25): V. Effect on Spatial Relations Abilities. *J. Psychol.* **39**, 435–442 (1955).

16. Abramson, H. A., Waxenberg, S. E., Levine, A., Kaufman, M. R. & Kornetsky, C. Lysergic Acid Diethylamide (Lsd-25) Xiii. Effect on Bender-Gestalt Test Performance. *J. Psychol.* **40**, 341–349 (1955).

17. Barrett, F. S., Carbonaro, T. M., Hurwitz, E., Johnson, M. W. & Griffiths, R. R. Double-blind comparison of the two hallucinogens psilocybin and dextromethorphan: effects on cognition. *Psychopharmacology (Berl.)* **235**, 2915–2927 (2018).

18. Heim, E., Heimann, H. & Lukács, G. Die psychische Wirkung der mexikanischen Droge „Ololiuqui“ am Menschen. *Psychopharmacologia* **13**, 35–48 (1968).

19. Hollister, L. E. Clinical, biochemical and psychologic effects of psilocybin. *Arch. Int. Pharmacodyn. Ther.* **130**, 42–52 (1961).

20. Hollister, L. E., Prusmack, J. J., Paulsen, J. A. & Rosenquist, N. COMPARISON OF THREE PSYCHOTROPIC DRUGS (PSILOCYBIN, JB-329, AND IT-290) IN VOLUNTEER SUBJECTS: *J. Nerv. Ment. Dis.* **131**, 428–434 (1960).

21. Hollister, L. E., Macnicol, M. F. & Gillespie, H. K. An hallucinogenic amphetamine analog (DOM) in man. *Psychopharmacologia* **14**, 62–73 (1969).

22. Netz, B., Jonsson, C.-O. & Bergqvist, S. EFFECTS OF LYSERGIC ACID DIETHYLAMIDE (LSD-25) ON NORMAL SUBJECTS IN A SCHIZOPHRENIA-DISCRIMINATING TEST BATTERY. *Scand. J. Psychol.* **4**, 143–148 (1963).

23. Weingartner, H., Snyder, S. H. & Faillace, L. A. DOM (STP), A New Hallucinogenic Drug: Specific Perceptual Changes. *J. Clin. Pharmacol. New Drugs* **11**, 103–111 (1971).

24. Snyder, S. H., Faillace, L. A. & Weingartner, H. DOM (STP), a new hallucinogenic drug, and DOET: effects in normal subjects. *Am. J. Psychiatry* **125**, 113–120 (1968).

25. Wilson, R. E. & Shagass, C. COMPARISON OF TWO DRUGS WITH PSYCHOTOMIMETIC EFFECTS (LSD AND DITRAN): *J. Nerv. Ment. Dis.* **138**, 277–286 (1964).

26. Levine, A., Abramson, H. A., Abramson, H. A. & Markham, S. Lysergic Acid Diethylamide (Lsd-25): Xvi. the Effect on Intellectual Functioning as Measured By the Wechsler-Bellevue Intelligence Scale. *J. Psychol.* **40**, 385–395 (1955).

27. Lienert, G. A. Mental age Regression Induced by Lysergic Acid Diethylamide. *J. Psychol.* **63**, 3–11 (1966).

28. Savage, C. Variations in ego feeling induced by D-lysergic acid diethylamide (LSD-25). *Psychoanal. Rev.* **42**, 1–16 (1955).

29. Safer, J. The effect of LSD on sleep-deprived men. *Psychopharmacologia* 414–424 (1970).

30. Gastaut, H., Ferrer, S., Castells, C., Lesevre, N. & Luschnat, K. Effect of the d-lysergic acid diethylamide on the psychic functions and on electroencephalogram. *Confin. Neurol.* **13**, 102–120 (1953).

31. Silverstein, A. B. Effects of Lysergic Acid Diethylamide (LSD-25) on Intellectual Functions. *Arch. Neurol. Psychiatry* **80**, 477 (1958).

32. Goldberger, L. Cognitive test performance under LSD-25, placebo and isolation. *J. Nerv. Ment. Dis.* **142**, 4–9 (1966).

33. Family, N. *et al.* Semantic activation in LSD: evidence from picture naming. *Lang. Cogn. Neurosci.* **31**, 1320–1327 (2016).

34. González, D., Torrens, M. & Farré, M. Acute Effects of the Novel Psychoactive Drug 2C-B on Emotions. *BioMed Res. Int.* **2015**, 1–9 (2015).

35. Brengelmann, J. C. Effects of LSD-25 on Tests of Personality. *J. Ment. Sci.* **104**, 1226–1236 (1958).

36. Landis, C. & Clausen, J. Certain Effects of Mescaline and Lysergic Acid on Psychological Functions. *J. Psychol.* **38**, 211–221 (1954).

37. Abramson, H. A., Jarvik, M. E. & Hirsch, M. W. Lysergic Acid Diethylamide (LSD-25): VII. Effect upon Two Measures of Motor Performance. *J. Psychol.* **39**, 455–464 (1955).

38. Parrott, A. C. & Lasky, J. Ecstasy (MDMA) effects upon mood and cognition: before, during and after a Saturday night dance. *Psychopharmacology (Berl.)* **139**, 261–268 (1998).

39. Kuypers, K. P. C. & Ramaekers, J. G. Acute dose of MDMA (75 mg) impairs spatial memory for location but leaves contextual processing of visuospatial information unaffected. *Psychopharmacology (Berl.)* **189**, 557–563 (2006).

40. Dumont, G. J. H. *et al.* Acute neuropsychological effects of MDMA and ethanol (co-)administration in healthy volunteers. *Psychopharmacology (Berl.)* **197**, 465–474 (2008).

41. Kuypers, K., Torre, R., Farre, M., Pujadas, M. & Ramaekers, J. Inhibition of MDMA ‐induced increase in cortisol does not prevent acute impairment of verbal memory. *Br. J. Pharmacol.* **168**, 607–617 (2013).

42. Marrone, G. F., Pardo, J. S., Krauss, R. M. & Hart, C. L. Amphetamine analogs methamphetamine and 3,4-methylenedioxymethamphetamine (MDMA) differentially affect speech. *Psychopharmacology (Berl.)* **208**, 169–177 (2010).

43. Hoshi, R., Pratt, H., Mehta, S., Bond, A. J. & Curran, H. V. An investigation into the sub-acute effects of ecstasy on aggressive interpretative bias and aggressive mood – are there gender differences? *J. Psychopharmacol. (Oxf.)* **20**, 291–301 (2006).

44. Agurto, C. *et al.* Detection of acute 3,4-methylenedioxymethamphetamine (MDMA) effects across protocols using automated natural language processing. *Neuropsychopharmacology* **45**, 823–832 (2020).

45. Dumont, G. *et al.* Acute psychomotor, memory and subjective effects of MDMA and THC co-administration over time in healthy volunteers. *J. Psychopharmacol. (Oxf.)* **25**, 478–489 (2011).

46. Kuypers, K. P. C., Wingen, M., Samyn, N., Limbert, N. & Ramaekers, J. G. Acute effects of nocturnal doses of MDMA on measures of impulsivity and psychomotor performance throughout the night. *Psychopharmacology (Berl.)* **192**, 111–119 (2007).

47. Lamers, C. T. J. *et al.* Dissociable Effects of a Single Dose of Ecstasy (MDMA) on Psychomotor Skills and Attentional Performance. *J. Psychopharmacol. (Oxf.)* **17**, 379–387 (2003).

48. Kuypers, K. P. C., Samyn, N. & Ramaekers, J. G. MDMA and alcohol effects, combined and alone, on objective and subjective measures of actual driving performance and psychomotor function. *Psychopharmacology (Berl.)* **187**, 467–475 (2006).

49. Prochazkova, L. *et al.* Exploring the effect of microdosing psychedelics on creativity in an open-label natural setting. *Psychopharmacology (Berl.)* **235**, 3401–3413 (2018).

50. Sanz, C. *et al.* Natural language signatures of psilocybin microdosing. 22.

51. Wießner, I. *et al.* LSD, afterglow and hangover: Increased episodic memory and verbal fluency, decreased cognitive flexibility. *Eur. Neuropsychopharmacol.* **58**, 7–19 (2022).

52. Ornelas, I. M. *et al.* Nootropic effects of LSD: Behavioral, molecular and computational evidence. *Exp. Neurol.* **356**, 114148 (2022).

53. Downing, J. The Psychological and Physiological Effects of MDMA on Normal Volunteers. *J. Psychoactive Drugs* **18**, 335–340 (1986).

54. Kirkpatrick, M. G. *et al.* A direct comparison of the behavioral and physiological effects of methamphetamine and 3,4-methylenedioxymethamphetamine (MDMA) in humans. *Psychopharmacology (Berl.)* **219**, 109–122 (2012).

55. Barendregt, J. T. Performance on Some Objective Tests under LSD-25. in *Advances in Psychosomatic Medicine* (eds. Jores, A., Freyberger, H. & Stokvis, B.) vol. 1 217–219 (S. Karger AG, 1959).

56. Resnick, O., Krus, D. M. & Raskin, M. LSD-25 action in normal subjects treated with a monoamine oxidase inhibitor. *Life Sci.* **3**, 1207–1214 (1964).

57. Resnick, O., Krus, D. M. & Raskin, M. ACCENTUATION OF THE PSYCHOLOGICAL EFFKTS OF LSD-25 IN NORMAL SUBJECTS TREATED WITH RESERPINE. *Life Sci.* 1433–1437 (1965).

58. Rynearson, R. R., Wilson, M. R. & Bickford, R. G. Psilocybin-induced changes in psychologic function, electroencephalogram, and light-evoked potentials in human subjects. *Mayo Clin. Proc.* **43**, 191–204 (1968).

59. Savage, C. Lysergic acid diethylamide (lsd-25). *Am. J. Psychiatry* **108**, 896–900 (1952).

60. Sjoerdsma, A., Kornetsky, C. & Evarts, E. Lysergic Acid Diethylamide in Patients withk Excess Serotonin. **Archives of Neurology and Psychiatry**, (1956).

61. Spitzer, M. *et al.* Increased activation of indirect semantic associations under psilocybin. *Biol. Psychiatry* **39**, 1055–1057 (1996).

62. Reckweg, J. *et al.* A Phase 1, Dose-Ranging Study to Assess Safety and Psychoactive Effects of a Vaporized 5-Methoxy-N, N-Dimethyltryptamine Formulation (GH001) in Healthy Volunteers. *Front. Pharmacol.* **12**, 760671 (2021).

63. Gamma, A. 3,4-Methylenedioxymethamphetamine (MDMA) Modulates Cortical and Limbic Brain Activity as Measured by [H215O]-PET in Healthy Humans. *Neuropsychopharmacology* **23**, 388–395 (2000).

64. Aronson, H., Watermann, C. E. & Klee, G. D. The effect of D-lysergic acid diethylamide (LSD-25) on learning and retention. *J. Clin. Exp. Psychopathol. Q. Rev. Psychiatry Neurol.* **23**, 17–23 (1962).

65. Cohen, B. D. Comparison of Phencyclidine Hydrochloride (Sernyl) with Other Drugs: Simulation of Schizophrenic Performance with Phencyclidine Hydrochloride (Sernyl), Lysergic Acid Diethylamide (LSD-25), and Amobarbital (Amytal) Sodium; II. Symbolic and Sequential Thinking. *Arch. Gen. Psychiatry* **6**, 395 (1962).

66. Rosenbaum, G. Comparison of Sernyl with Other Drugs: Simulation of Schizophrenic Performance with Sernyl, LSD-25, and Amobarbital (Amytal) Sodium; I. Attention, Motor Function, and Proprioception. *AMA Arch. Gen. Psychiatry* **1**, 651 (1959).

67. Abramson, H. A., Jarvik, M. E. & Hirsch, M. W. Lysergic Acid Diethylamide (Lsd-25): X. Effect on Reaction Time to Auditory and Visual Stimuli. *J. Psychol.* **40**, 39–52 (1955).

68. Jarvik, M. E., Abramson, H. A. & Hirsch, M. W. Lysergic Acid Diethylamide (LSD-25): VI. Effect upon Recall and Recognition of Various Stimuli. *J. Psychol.* **39**, 443–454 (1955).

69. Jarvik, M. E., Abramson, H. A. & Hirsch, M. W. Lysergic Acid Diethylamide (LSD-25): IV. Effect on Attention and Concentration. *J. Psychol.* **39**, 373–383 (1955).

70. Ostfeld, A. M. Effects of LSD 25 and JB 318 on tests of visual and perceptual functions in man. *Fed. Proc.* **20**, 876–884 (1961).

71. Pirona, A. & Morgan, M. An investigation of the subacute effects of ecstasy on neuropsychological performance, sleep and mood in regular ecstasy users. *J. Psychopharmacol. (Oxf.)* **24**, 175–185 (2010).

72. Dumont, G. *et al.* Acute psychomotor effects of MDMA and ethanol (co-) administration over time in healthy volunteers. *J. Psychopharmacol. (Oxf.)* **24**, 155–164 (2010).

73. Haijen, E. *et al.* Peripheral endocannabinoid concentrations are not associated with verbal memory impairment during MDMA intoxication. *Psychopharmacology (Berl.)* **235**, 709–717 (2018).

74. Hasler, F., Studerus, E., Lindner, K., Ludewig, S. & Vollenweider, F. Investigation of serotonin-1A receptor function in the human psychopharmacology of MDMA. *J. Psychopharmacol. (Oxf.)* **23**, 923–935 (2009).

75. Hysek, C. M., Domes, G. & Liechti, M. E. MDMA enhances “mind reading” of positive emotions and impairs “mind reading” of negative emotions. *Psychopharmacology (Berl.)* **222**, 293–302 (2012).

76. Kuypers, K. P. C. & Ramaekers, J. G. Transient memory impairment after acute dose of 75mg 3.4-Methylene-dioxymethamphetamine. *J. Psychopharmacol. Oxf. Engl.* **19**, 633–639 (2005).

77. Schmidt, A. *et al.* Comparative Effects of Methylphenidate, Modafinil, and MDMA on Response Inhibition Neural Networks in Healthy Subjects. *Int. J. Neuropsychopharmacol.* **20**, 712–720 (2017).

78. van Wel, J. H. P. *et al.* Blockade of 5-HT2 Receptor Selectively Prevents MDMA-Induced Verbal Memory Impairment. *Neuropsychopharmacology* **36**, 1932–1939 (2011).

79. Gouzoulis-Mayfrank, E. *et al.* Effects of the Hallucinogen Psilocybin on Covert Orienting of Visual Attention in Humans. *Neuropsychobiology* **45**, 205–212 (2002).

80. Vollenweider, F. X., Vollenweider-Scherpenhuyzen, M. F. I., Bäbler, A., Vogel, H. & Hell, D. Psilocybin induces schizophrenia-like psychosis in humans via a serotonin-2 agonist action: *NeuroReport* **9**, 3897–3902 (1998).

81. Gouzoulis-Mayfrank, E. *et al.* Inhibition of Return in the Human 5HT2A Agonist and NMDA Antagonist Model of Psychosis. *Neuropsychopharmacology* **31**, 431–441 (2006).

82. Harman, W. W., Mckim, R. H., Mogar, R. E. & Fadiman, J. PSYCHEDELIC AGENTS IN CREATIVE PROBLEM-SOLVING: A PILOT. *Psychol. Rep.* **19**, 211–227 (1966).

83. Heekeren, K. *et al.* Mismatch negativity generation in the human 5HT2A agonist and NMDA antagonist model of psychosis. *Psychopharmacology (Berl.)* **199**, 77–88 (2008).

84. Heekeren, K. *et al.* Prepulse inhibition of the startle reflex and its attentional modulation in the human S-ketamine and N,N-dimethyltryptamine (DMT) models of psychosis. *J. Psychopharmacol. (Oxf.)* **21**, 312–320 (2007).

85. Kanen, J. W. *et al.* Effect of lysergic acid diethylamide (LSD) on reinforcement learning in humans. *Psychol. Med.* 1–12 (2022) doi:10.1017/S0033291722002963.

86. Kometer, M. *et al.* Psilocybin Biases Facial Recognition, Goal-Directed Behavior, and Mood State Toward Positive Relative to Negative Emotions Through Different Serotonergic Subreceptors. *Biol. Psychiatry* **72**, 898–906 (2012).

87. Ramaekers, J. *et al.* Altered State of Consciousness and Mental Imagery as a Function of *N* , *N* -dimethyltryptamine Concentration in Ritualistic Ayahuasca Users. *J. Cogn. Neurosci.* 1–12 (2023) doi:10.1162/jocn_a_02003.

88. Thatcher, K., Wiederholt, W. C. & Fischer, R. An electroencephalographic analysis of personality-dependent performance under psilocybin. *Agents Actions* **2**, 21–26 (1971).

89. Umbricht, D. *et al.* Effects of the 5-HT2A Agonist Psilocybin on Mismatch Negativity Generation and AX-Continuous Performance Task: Implications for the Neuropharmacology of Cognitive Deficits in Schizophrenia. *Neuropsychopharmacology* **28**, 170–181 (2003).

90. Vollenweider, F. X., Csomor, P. A., Knappe, B., Geyer, M. A. & Quednow, B. B. The Effects of the Preferential 5-HT2A Agonist Psilocybin on Prepulse Inhibition of Startle in Healthy Human Volunteers Depend on Interstimulus Interval. *Neuropsychopharmacology* **32**, 1876–1887 (2007).

91. Wit, H., Molla, H. M., Bershad, A., Bremmer, M. & Lee, R. Repeated low doses of LSD in healthy adults: A placebo‐controlled, dose–response study. *Addict. Biol.* **27**, (2022).

92. Fallon, S. J. *No Evidence That LSD Microdosing Affects Recall or the Balance between Distracter Resistance and Updating*. http://biorxiv.org/lookup/doi/10.1101/2021.12.02.470935 (2021) doi:10.1101/2021.12.02.470935.

93. Hutten, N. R. P. W. *et al.* Low Doses of LSD Acutely Increase BDNF Blood Plasma Levels in Healthy Volunteers. *ACS Pharmacol. Transl. Sci.* **4**, 461–466 (2021).

94. Mason, N. L., Mischler, E., Uthaug, M. V. & Kuypers, K. P. C. Sub-Acute Effects of Psilocybin on Empathy, Creative Thinking, and Subjective Well-Being. *J. Psychoactive Drugs* **51**, 123–134 (2019).

95. Uthaug, M. V. *et al.* Sub-acute and long-term effects of ayahuasca on affect and cognitive thinking style and their association with ego dissolution. *Psychopharmacology (Berl.)* **235**, 2979–2989 (2018).

96. Uthaug, M. V. *et al.* A single inhalation of vapor from dried toad secretion containing 5-methoxy-N,N-dimethyltryptamine (5-MeO-DMT) in a naturalistic setting is related to sustained enhancement of satisfaction with life, mindfulness-related capacities, and a decrement of psychopathological symptoms. *Psychopharmacology (Berl.)* **236**, 2653–2666 (2019).

97. Wießner, I. *et al.* LSD and creativity: Increased novelty and symbolic thinking, decreased utility and convergent thinking. *J. Psychopharmacol. (Oxf.)* **36**, 348–359 (2022).

98. Bouso, J. C., Fábregas, J. M., Antonijoan, R. M., Rodríguez-Fornells, A. & Riba, J. Acute effects of ayahuasca on neuropsychological performance: differences in executive function between experienced and occasional users. *Psychopharmacology (Berl.)* **230**, 415–424 (2013).

99. Dittrich, A. Alteration of behavioural changes induced by 3,4,5-trimethoxyphenylethylamine (mescaline) by pretreatment with 2,4,5-trimethoxyphenylethylamine: A self-experiment. *Psychopharmacologia* **21**, 229–237 (1971).

100. Zegans, L. S. The Effects of LSD-25 on Creativity and Tolerance to Regression. *Arch. Gen. Psychiatry* **16**, 740 (1967).

101. Kornetsky, C. Comparison of Psychological Effects of Certain Centrally Acting Drugs in Man. *Arch. Neurol. Psychiatry* **77**, 318 (1957).

102. Primac, D. W. Effects of Centrally Acting Drugs on Two Tests of Brain Damage. *Arch. Neurol. Psychiatry* **77**, 328 (1957).

103. Sloane, B. & Doust, J. W. L. Psychophysiological Investigations in Experimental Psychoses: Results of the Exhibition of D-Lysergic Acid Diethylamide to Psychiatric Patients. *J. Ment. Sci.* **100**, 129–144 (1954).

104. Wapner, S. & Krus, D. M. Effects of lysergic acid diethylamide, and differences between normals and schizophrenics on the Stroop Color-Word Test. *J. Neuropsychiatry* **2**, 76–81 (1960).

105. Doss, M. K. *et al.* Psilocybin therapy increases cognitive and neural flexibility in patients with major depressive disorder. *Transl. Psychiatry* **11**, 574 (2021).

106. Mason, N. L. *et al.* Spontaneous and deliberate creative cognition during and after psilocybin exposure. *Transl. Psychiatry* **11**, 1–13 (2021).

107. Kuypers, K. P. C. *et al.* Ayahuasca enhances creative divergent thinking while decreasing conventional convergent thinking. *Psychopharmacology (Berl.)* **233**, 3395–3403 (2016).

108. Family, N. *et al.* Safety, tolerability, pharmacokinetics, and pharmacodynamics of low dose lysergic acid diethylamide (LSD) in healthy older volunteers. *Psychopharmacology (Berl.)* **237**, 841–853 (2020).

109. Yanakieva, S. *et al.* The effects of microdose LSD on time perception: a randomised, double-blind, placebo-controlled trial. *Psychopharmacology (Berl.)* **236**, 1159–1170 (2019).

110. Kiraga, M. K. *et al.* Persisting Effects of Ayahuasca on Empathy, Creative Thinking, Decentering, Personality, and Well-Being. *Front. Pharmacol.* **12**, 721537 (2021).

111. Murphy-Beiner, A. & Soar, K. Ayahuasca’s ‘afterglow’: Improved mindfulness and cognitive flexibility in ayahuasca drinkers. *Psychopharmacology (Berl.)* **237**, 1161–1169 (2020).
